# CowN sustains nitrogenase turnover in the presence of the inhibitor carbon monoxide

**DOI:** 10.1016/j.jbc.2021.100501

**Published:** 2021-03-02

**Authors:** Michael S. Medina, Kevin O. Bretzing, Richard A. Aviles, Kiersten M. Chong, Alejandro Espinoza, Chloe Nicole G. Garcia, Benjamin B. Katz, Ruchita N. Kharwa, Andrea Hernandez, Justin L. Lee, Terrence M. Lee, Christine Lo Verde, Max W. Strul, Emily Y. Wong, Cedric P. Owens

**Affiliations:** 1 Schmid College of Science and Technology, Chapman University, Orange, California, USA; 2 Department of Chemistry, University of California, Irvine, Irvine, California, USA

**Keywords:** carbon monoxide, *Gluconacetobacter diazotrophicus*, iron–sulfur cluster, iron–sulfur protein, nitrogen fixation, nitrogenase, protein–protein interaction, DLS, dynamic light scattering, EDC, (1-ethyl-3-(3-dimethylaminopropyl)carbodiimide hydrochloride), FeMoco, iron–molybdenum cofactor, FeP, iron–protein, FeVco, iron–vanadium cofactor, ICP-OES, inductively coupled plasma–optical emission spectroscopy, MBP, maltose binding protein, MoFeP, molybdenum–iron protein, NHS, N-hydroxysuccinamide, RT-qPCR, reverse transcription quantitative polymerase chain reaction, TCEP, (tris(2-carboxyethyl)phosphine), UAS, upstream activator sequence, VFeP, vanadium–iron protein

## Abstract

Nitrogenase is the only enzyme capable of catalyzing nitrogen fixation, the reduction of dinitrogen gas (N_2_) to ammonia (NH_3_). Nitrogenase is tightly inhibited by the environmental gas carbon monoxide (CO). Nitrogen-fixing bacteria rely on the protein CowN to grow in the presence of CO. However, the mechanism by which CowN operates is unknown. Here, we present the biochemical characterization of CowN and examine how CowN protects nitrogenase from CO. We determine that CowN interacts directly with nitrogenase and that CowN protection observes hyperbolic kinetics with respect to CowN concentration. At a CO concentration of 0.001 atm, CowN restores nearly full nitrogenase activity. Our results further indicate that CowN’s protection mechanism involves decreasing the binding affinity of CO to nitrogenase’s active site approximately tenfold without interrupting substrate turnover. Taken together, our work suggests CowN is an important auxiliary protein in nitrogen fixation that engenders CO tolerance to nitrogenase.

Biological nitrogen fixation, the reduction of dinitrogen gas (N_2_) to ammonia (NH_3_), is catalyzed exclusively by the bacterial metalloenzyme nitrogenase in an ATP-dependent process (Equation 1).

(1) $$N_{2\;}+16\;ATP+8\;e^{-}+8H^{+}\to 2NH_{3}+H_{2}+16ADP+16P_{i}$$

There are three types of nitrogenases that differ by their active site metal cluster composition. All nitrogen-fixing bacteria (diazotrophs) contain molybdenum-nitrogenase (Mo-nitrogenase) (1, 2). Mo-nitrogenase consists of the catalytic molybdenum–iron protein (MoFeP) and its reductase, iron–protein (FeP) (Fig. 1*A*) (3, 4). MoFeP contains two unique metal clusters, the [Mo:7Fe:9S:1C] active site, referred to as FeMoco, and the [8Fe:7S] P-cluster. FeP shuttles electrons from its [4Fe:4S] cluster to MoFeP in an ATP-dependent reaction (Equation 1) (5, 6). In addition to Mo-nitrogenase, a subset of diazotrophs contain one or both of the alternative nitrogenases, Vanadium-nitrogenase (V-nitrogenase) and Iron-only nitrogenase (Fe-nitrogenase) (2). These nitrogenases contain a V or Fe metal instead of Mo in their respective active sites. V-nitrogenase further differs structurally from Mo-nitrogenase in that it contains a small α-helical subunit, the δ-subunit, which binds to VFeP (the catalytic component of V-nitrogenase) in the vicinity of the active site (Fig. S1*A*) (7, 8, 9).

**Figure 1:**
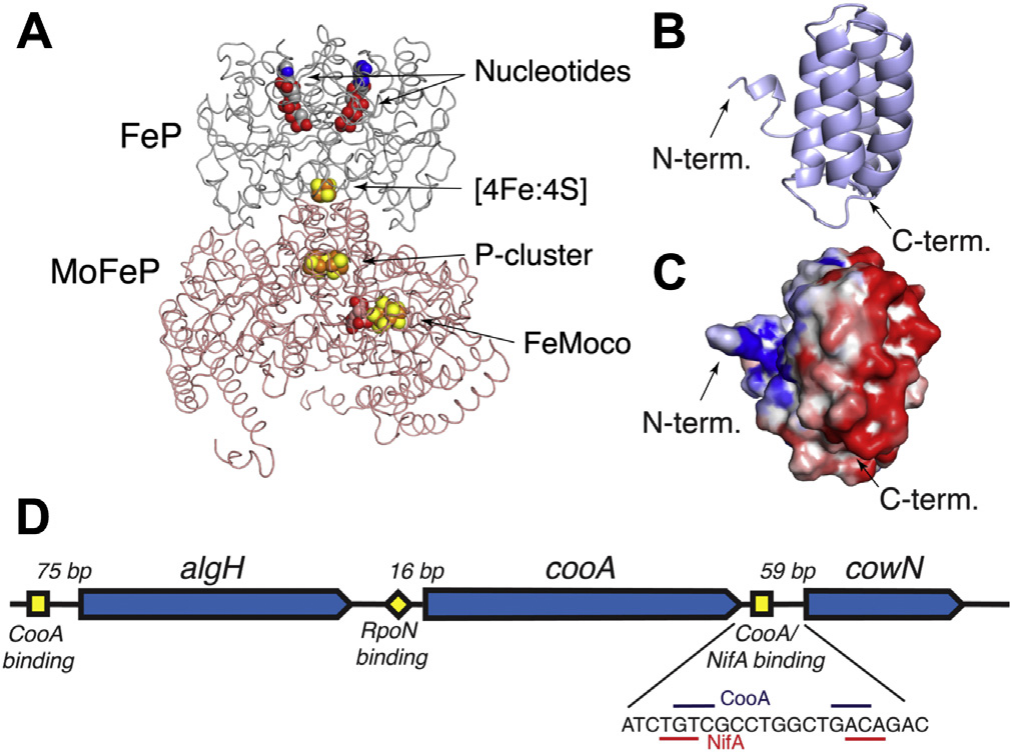
**Structure of nitrogenase, CowN model, and genomic region.***A*, structure of nitrogenase (PDB: 4WZB). MoFeP is colored *pink* and FeP is *gray*. Nitrogenase’s metal clusters and nucleotides are shown as *spheres*. *B*, structural model of CowN, generated by I-tasser (60) showing the predicted 4-helical bundle fold. *C*, surface electrostatic potential based on CowN model, calculated using Bluues (61) where negatively and positively charged residues are colored *red* and *blue*, respectively. *D*, genomic region surrounding CowN in *G. diazotrophicus*. Open reading frames are shown as *arrows*. Predicted upstream activator sequences are shown as *yellow squares* and the predicted RpoN binding site is shown as a *diamond*.

In addition to N_2_, nitrogenase is able to reduce other substrates, including acetylene (C_2_H_2_) and protons (H^+^) (10). All substrate reduction except for H^+^ reduction is inhibited by the abundant environmental gas carbon monoxide (CO) in all three types of nitrogenase in either a noncompetitive or mixed fashion (11, 12, 13). CO is generated both anthropogenically (https://www.epa.gov/sites/production/files/2017-04/documents/2014neiv1_profile_final_april182017.pdf, accessed May 29, 2020) and naturally by soil chemical processes (14, 15). CO has also been identified as a plant signaling molecule that plays a role in lateral root formation (16, 17, 18).

Unsurprisingly, diazotrophs have evolved defenses to prevent inhibition of nitrogen fixation by CO. Protection against CO is best described in *Rhodospirillum rubrum* (19) and *Rhodobacter* *capsulatus* (20). Both organisms are able to grow diazotrophically in the presence of CO. These results are surprising since CO inhibition of nitrogenase is expected to prevent bacterial growth. Protection against CO inhibition hinges on a protein called CowN. Knocking out CowN in either *R. rubrum* or *R. capsulatus* renders the bacteria incapable of growing diazotrophically under CO (19, 20).

Although CO is a potent inhibitor of N_2_ reduction, it is also a nitrogenase substrate that is converted into hydrocarbons (21, 22). V-nitrogenase has the highest CO reducing activity; it is about 800-fold more active toward CO compared with Mo-nitrogenase (23, 24). CowN exclusively sustains Mo-nitrogenase-dependent growth, as CowN expression is not triggered under diazotrophic growth supported by alternative nitrogenases (20). The reason for this is unclear; however, it has been suggested that alternative nitrogenases do not require the action of CowN since they may circumvent CO inhibition by reducing CO to hydrocarbons (20).

While the role of CowN as a CO-protective protein is established, its mechanism is unknown. We do not know if CowN acts directly on nitrogenase. It is possible that CowN’s target is not nitrogenase but another vital part of the bacterial nitrogen-fixing machinery that is susceptible to inactivation by CO, such as the *nuo* electron transport system (25) or protective terminal oxidases (26).

Previous studies on CowN did not measure nitrogenase activity under diazotrophic growth conditions in the presence of CO. On the one hand, it has been shown that CO inhibits N_2_ reduction *in vivo* (11). On the other, the fact that *R. rubrum* and *R. capsulatus* growth kinetics were not significantly slowed by CO would suggest that nitrogenase must remain active (19, 20). Nevertheless, it was proposed that CowN acts similarly to Shethna protein. Shethna protein protects nitrogenase from O_2_ oxidation by locking FeP and MoFeP together to block O_2_ access to nitrogenase’s oxygen-sensitive metal clusters (27, 28). A similar mechanism for CowN could either involve trapping nitrogenase in a conformation that blocks CO binding to the active site or prevent FeMoco from reaching the E2 oxidation state required for CO binding (29).

CowN is structurally uncharacterized, but is predicted to be a four-helical bundle (Fig. 1*B*). It does not feature a known cofactor binding site, nor is it predicted to coordinate metals. CowN is therefore unlikely to react with CO or take part in CO transport. Instead, we postulate that CowN exerts its protective effect through protein–protein interactions with nitrogenase. CowN is predicted to have a negative surface charge profile (Fig. 1*C*) that is complementary to positive patches on the MoFeP surface near FeMoco and known CO access channels (Fig. S1, *B* and *C*). This raises the possibility that CowN binds to MoFeP in a similar manner as δ-subunit binds to VFeP, close to the active site, where CowN could influence FeMoco reactivity and substrate binding. Alternatively, CowN may bind at the entrance of a substrate channel and thereby alter gas access. Based on our current knowledge of CowN, two hypotheses emerge on how CowN prevents CO inhibition of nitrogenase:

(1) CowN operates similarly to Shethna protein and protects nitrogenase by shutting down turnover.
(2) CowN prevents CO binding to nitrogenase without shutting down turnover. In this case, nitrogenase turns over normally in the presence of CowN since CO is selectively prevented from tightly binding to FeMoco.

A third potential mechanism is that CowN engenders substantial CO reduction activity to Mo-nitrogenase. While intriguing, this possibility is unlikely since extensive *in vivo* experiments detected only negligible hydrocarbon formation by Mo-nitrogenase under CO (24).

To examine our hypotheses, we have expressed and purified recombinant CowN and determined its effect on nitrogenase in the presence of CO. Our results suggest CowN interacts directly with nitrogenase. CowN does not shut down nitrogenase. Instead, CowN enables nitrogenase to turn over under CO by significantly increasing the inhibition constant of CO.

## Results and discussion

### Genomic organization around CowN

We chose to study CowN in the diazotroph *Gluconacetobacter diazotrophicus*, an agriculturally relevant organism with a well-characterized nitrogenase (30, 31, 32). Importantly, *G. diazotrophicus* has no alternative nitrogenases so any regulatory cross talk between nitrogenases that may influence CowN expression can be ruled out. In *G. diazotrophicus*, CowN is part of a three-gene cluster consisting of *algH*, *cooA*, and *cowN* (Fig. 1*D*). *CooA* is a CO-responsive hemoprotein (33) that activates gene expression by binding to a tGTCg-(N)_6_-tGACa (lower case letters denote less conserved nucleotides) upstream activator sequence (UAS) (19). This sequence is found 60 base pairs (bp) upstream of *cowN* and 75 bp ahead of *algH*. This indicates that CowN is likely regulated by CooA, consistent with regulation in *R. capsulatus* (20). The functional importance of CooA regulation of *algH* is unclear. *AlgH* is a regulatory protein with an α/β structure of unknown function (34) and *algH* is not found in the genomic vicinity of CowN in many diazotrophs. CowN expression is also dependent on the central nitrogenase regulator NifA, which binds to a TGT-(N)_10_-ACA UAS (35) 59 bp upstream of CowN. The overlap between the CooA and NifA UAS suggests there is interplay between CooA and NifA in regulating CowN expression. CooA expression in *G. diazotrophicus* is likely dependent on RpoN, a σ^54^ factor commonly associated with nitrogen fixation (36).

### Carbon monoxide and nitrogen fixation conditions increase CowN expression

**Figure 2:**
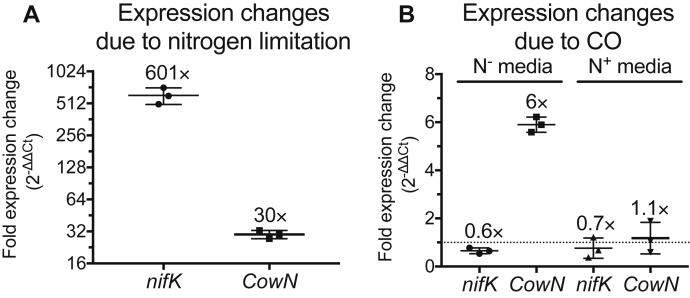
**Changes in *cowN* and *nifK* (MoFeP β-subunit) expression measured by RT-qPCR.***A*, expression changes due to diazotrophic growth. *B*, expression changes due to CO. The *boxes* represent the data range and *middle line* the mean. The *dotted line* in *B* indicates 1-fold expression (*i.e.*, no change in expression). *NifK* in panel *A* serves as a positive control. Its expression is expected to be turned on in nitrogen-limited media. In panel *B*, *nifK* expression is slightly reduced *versus* N^−^ media without CO; however, the change is small and nitrogenase is still highly expressed. Results of triplicate experiments are shown. The different y-axis in *A* and *B* reflects the different magnitude of expression change.

Previous studies in diazotrophs that have both Mo- and alternative nitrogenases indicate that CowN expression is turned on by CO and, to a lesser extent, nitrogen fixing conditions (20). To examine how CowN expression is regulated in *G. diazotrophicus*, which lacks alternative nitrogenases, we performed RT-qPCR assays using bacterial cultures grown either diazotrophically or with abundant (NH_4_)_2_SO_4_. As indicated in Figure 2*A*, CowN expression increases 30-fold under diazotrophic conditions when compared with cultures that are given extra NH_4_^+^. Expression is induced to an even greater extent under diazotrophic conditions in the presence of CO (Fig. 2*B*, N^−^ media). CowN expression is specific to diazotrophic growth and not enhanced under CO in (NH_4_)_2_SO_4_ replete cultures (Fig. 2*B*, N^+^ media). Our results follow the same pattern seen in *R. capsulatus* (20), indicating that the CowN response to CO is fundamentally the same between diazotrophs that harbor exclusively Mo-nitrogenase and those that have alternative nitrogenases. Furthermore, our results confirm that CowN is expressed under all nitrogen fixing conditions, suggesting CowN may have an unidentified role in maintaining nitrogen fixation in the absence of exogenous CO. Alternatively, CowN may be expressed in response to very low levels of metabolic CO production, known to occur in some diazotrophs (37).

### Expression and purification of CowN

CowN’s mechanism for sustaining nitrogen fixation under CO is unknown. To generate CowN for functional assays with nitrogenase, we cloned *cowN* into a pET28a expression plasmid and expressed the protein heterologously as a His-tagged fusion protein in *Escherichia coli*. Recombinant His-CowN expressed in inclusion bodies. Attempts to obtain soluble protein by changing growth media and temperature were unsuccessful (Fig. S2). A second construct was made where CowN was expressed and purified as a Maltose Binding Protein (MBP) fusion since the soluble MBP tag is known to help prevent inclusion body formation (Fig. S3*A*) (38). However, MBP-CowN mostly formed a high-molecular-weight soluble aggregate, suggesting that CowN (Fig. S3*B*), when expressed recombinantly, has the propensity to aggregate.

**Figure 3:**
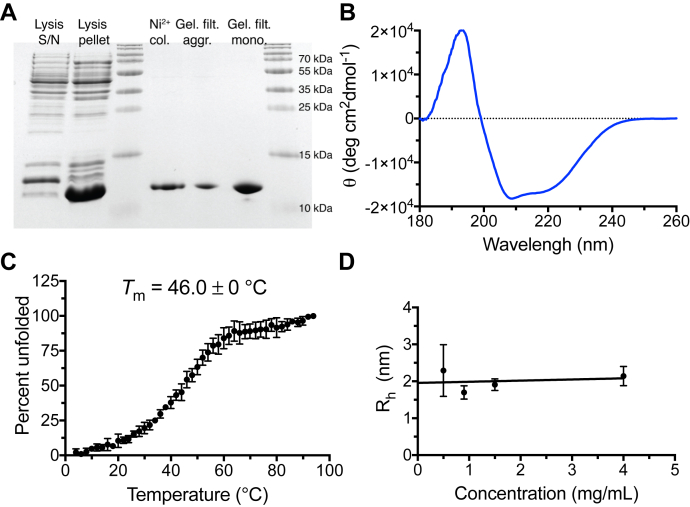
**Purification and physical properties of CowN.***A*, SDS-PAGE of His-CowN in different stages of purification. *B*, CD spectrum of CowN showing predominantly α-helical secondary structure. *C*, thermal denaturation curve of CowN. *D*, determination of CowN radius by DLS, where the y-axis intercept represents the concentration independent radius.

To obtain CowN for nitrogenase experiments, we used His-CowN. His-CowN (henceforth referred to as CowN) was solubilized with urea and refolded after Ni^2+^ purification through stepwise dialysis. CowN was then purified to homogeneity by gel filtration chromatography (Fig. 3*A*), leading to the separation of aggregated from monomeric CowN (Fig. S4*A*). The molecular weight of CowN was confirmed by MALDI-TOF mass spectrometry (Fig. S5). Analysis by CD spectroscopy indicates that aggregated CowN represents misfolded protein (Fig. S6). Monomeric CowN is α-helical (Fig. 3*B*), consistent with structural predictions (Fig. 1*B*). CowN’s thermal stability was measured by monitoring unfolding at 222 nm using CD spectroscopy (Fig. 3*C*). Its *T*_m_ is equal to 46.0 °C, indicating CowN is folded in the temperature range in which *G. diazotrophicus* nitrogenase operates (around 30.0 °C). Cleavage of the His-tag resulted in immediate precipitation of CowN, suggesting that the tag is needed to keep the protein in solution. As discussed below, the His-tag does not appear to interfere with CowN activity.

The size of CowN based on gel filtration is 19.4 kDa (Fig. S4, *B* and *C*), lying between that of a CowN monomer (13 kDa) and dimer (26 kDa). Dynamic light scattering (DLS) experiments reveal that CowN has a hydrodynamic radius of 1.96 ± 0.25 nm, from which a 15 to 18 kDa molecular weight can be calculated (Fig. 3*D*). These experiments suggest CowN is most likely monomeric. CowN has a single Cys residue that can potentially form intermolecular disulfide bonds. Refolding the protein in the presence of DTT and performing gel filtration chromatography under reducing conditions does not increase the amount of CowN monomer, nor does it change the retention time of the monomer peak or CowN’s secondary structure (Figs. S5D and S6), suggesting that CowN does not form intermolecular disulfide bonds with itself.

### *G. diazotrophicus* nitrogenase purification

Nitrogenase was expressed and purified based on previously described methods (Fig. S7) (30). Our typical maximum MoFeP C_2_H_2_ reduction activity is about 1500 nmol mg^−1^ min^−1^ (Fig. S8), slightly higher than in previous reports (31, 32). The integrity of MoFeP and FeP metal clusters was confirmed by EPR (Fig. S9). The EPR spectrum of *G. diazotrophicus* FeP, reported here for the first time, is nearly identical to that of *Azotobacter vinelandii* FeP (39) and features an S = 1/2 signal in its ATP-bound form.

### CowN protects nitrogenase from inhibition by CO without shutting down turnover

**Figure 4:**
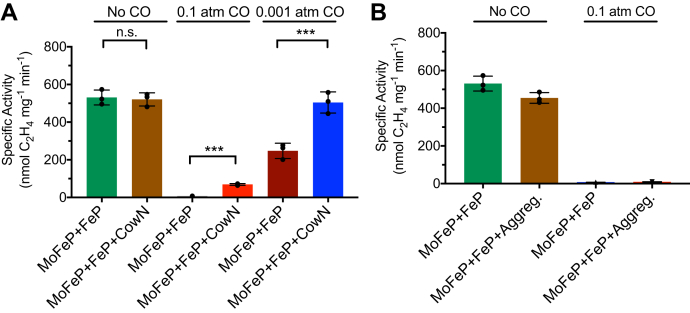
**Effect of CowN on nitrogenase activity.***A*, nitrogenase C_2_H_2_ reduction in the presence and absence of CowN with no CO, 0.1 atm CO, or 0.001 atm CO. *B*, control experiments determining that unfolded CowN does not protect nitrogenase. MoFeP and FeP concentrations are 0.2 μM and 2 μM, respectively. In *A* and *B*, the CowN concentration is 2 μM. Activity differences that are significant to *p* = 0.001 are denoted by ∗∗∗. n.s. means no statistical difference.

We first tested the hypothesis that CowN operates similar to Shethna protein. In such a case, we expected CowN to turn off nitrogenase activity, as measured using reduction of the established nitrogenase substrate acetylene (C_2_H_2_) to ethylene (C_2_H_4_) (40). As shown in Figure 4*A* and Fig. S10, nitrogenase activity is the same in the presence and absence of CowN. These data suggest that CowN does not stop nitrogenase turnover and must have a different mechanism compared with Shethna protein. This result is consistent with diazotrophs’ ability to grow efficiently under CO, as discussed earlier (19, 20).

We next tested whether CowN allows nitrogenase to remain catalytically active under CO (Fig. 4*A*). In the absence of CowN, nitrogenase is nearly completely inhibited under 0.1 atm CO and 50 to 60% inhibited under 0.001 atm. With the addition of CowN, nitrogenase activity increases about ninefold for samples containing 0.1 atm CO. Strikingly, nitrogenase activity under 0.001 atm CO is nearly fully restored by CowN.

Control experiments with unfolded CowN aggregate (from the 8.3 ml peak in Fig. S4*A*) demonstrate that unfolded CowN does not prevent CO inhibition of nitrogenase (Fig. 4*B* and Fig. S10). Likewise, addition of BSA, which binds to proteins nonspecifically, does not protect nitrogenase from CO inhibition (Fig. S10). These results suggest CowN likely interacts with nitrogenase through a specific interaction. To determine if CowN’s His-tag interferes with its activity, we performed CO protection experiments with nonaggregated MBP-CowN (14.2 ml peak in Fig. S3*B*). We found similar results between His-CowN and MBP-CowN (Fig. S11), indicating the tag does not interfere with CowN activity. Further control experiments suggest that CowN, by itself, does not reduce either CO or C_2_H_2_ (Fig. S12). We also confirmed that CowN does not promote CO reduction by Mo-nitrogenase (Fig. S12), as expected based on earlier studies (24).

### Background on CO binding to MoFeP

We present a brief summary of the mechanism of CO binding to MoFeP since this will help contextualize results that are presented in the subsequent sections. CO inhibition kinetics are described by Equation 2.

(2) $$v_{o}=\;\frac{V_{max}\left[S\right]}{\alpha K_{M}+\alpha^{\prime }\left[S\right]}\;$$

where α and α’ represent the strength of inhibitor binding to the free enzyme and the enzyme–substrate complex, respectively. α and α’ are given by:

$$\alpha =1+\;\frac{\left[I\right]}{K_{Ia}}$$

$$\alpha^{\prime }=1+\;\frac{\left[I\right]}{K_{Ib}}$$

*K*_Ia_ is the inhibition constant for inhibitor binding to the free enzyme and *K*_Ib_ is the inhibition constant for binding to the ES complex. In pure noncompetitive inhibition, *K*_Ia_ and *K*_Ib_ are equal to each other, whereas *K*_Ia_ ≠ *K*_Ib_ for a mixed inhibitor. For a competitive inhibitor *K*_Ia_ << *K*_Ib_ and for an uncompetitive inhibitor *K*_Ia_ >> *K*_Ib_.

CO inhibits most nitrogenases in a mixed fashion (11, 12, 13, 41) (Mixed inhibition is referred to as noncompetitive when using Cleland’s classification). CO most likely acts as a mixed inhibitor by binding to more than one site on or near FeMoco, thereby both influencing substrate binding to the free enzyme and inhibiting turnover of the ES complex. In the mixed case, *K*_Ia_ is 1 to 2 × 10^−4^ atm, whereas *K*_Ib_ is about 2- to 5-fold higher (12, 13). Several CO-bound nitrogenase intermediates have been characterized spectroscopically (42, 43, 44, 45) and by X-ray crystallography (46, 47). However, we caution against trying to assign *K*_Ia_ and *K*_Ib_ based on a particular CO-bound structure since the number of known CO-bound forms of MoFeP exceeds the number of inhibition constants and we do not know which CO bound structure is relevant for a particular inhibition mode. CO acts as a mixed inhibitor by directly binding to FeMoco (46, 47) and/or CO may compete for binding sites along substrate channels that lead to FeMoco (47). One of the potential CO access routes has been confirmed by mutagenesis experiments. When residue αG69 (*A. vinelandii* numbering) is mutated to Ser, CO becomes a competitive inhibitor, leading to the conclusion that CO reaches FeMoco *via* αG69 (12). αG69 is located at the terminus of the so-called Igarashi channel (also referred to as IS channel) that starts on the protein surface at residues αK176 and αE263 and later passes by αV70 (*A. vinelandii* numbering) (5, 48). Subsequently, IR spectroscopy experiments and MD simulations provided further evidence that CO likely migrates through this channel (49).

### CowN protects nitrogenase by decreasing the CO-binding affinity

The simplest explanation for CowN’s mechanism is that it lowers the affinity of CO binding to FeMoco. To test this hypothesis, we measured the Michaelis–Menten constant, *K*_M_, of substrate binding to MoFeP and the inhibition constant, *K*_I_, for CO in the absence of CowN and in the presence of 2 μM CowN. The *K*_M_ of *G. diazotrophicus* nitrogenase toward the substrate C_2_H_2_ is 0.0061 ± 0.0028 atm without CowN and 0.0068 ± 0.0023 atm with CowN (Table 1). These values are in good agreement with those reported for C_2_H_2_ binding to *A. vinelandii* nitrogenase (12). Since the *K*_M_ is not altered by CowN, we rule out that CowN selectively increases the affinity of nitrogenase for its substrates.

**Table 1:** Kinetic constants for C_2_H_2_ binding and CO inhibition

Protein	*K*_M_ for C_2_H_2_ binding (atm)	*K*_I_ for CO binding (atm) Mixed model	*K*_I_ for CO binding (atm) Competitive model
*Gd*-nitrogenase	6.11 ± 2.79 × 10^−3^	*K*_Ia_ = 1.4 ± 0.4 × 10^−4^	*K*_I_ = 1.1 ± 0.3 × 10^−4^
		*K*_Ib_ = 8.97 ± 8.47 × 10^−3^	
*Gd*-nitrogenase + CowN	6.81 ± 2.28 × 10^−3^	*K*_Ia_ = 1.43 ± 1.04 × 10^−3^	*K*_I_ = 6.2 ± 3.2 × 10^−4^
		*K*_Ib_ = 1.523 ± 1.226 × 10^−2^	

**Figure 5:**
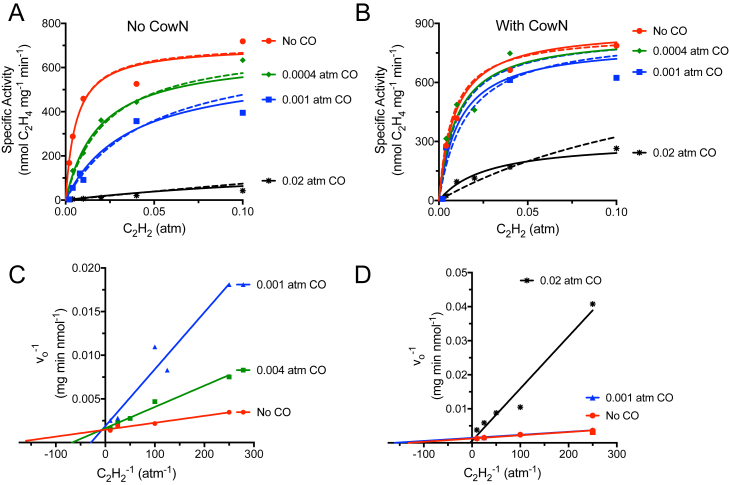
**Inhibition kinetics of CO binding to MoFeP.***A*, CO binding in absence of CowN and *B*, in the presence of CowN. MoFeP, FeP, and CowN concentrations are 0.2 μM, 2 μM, and 2 μM, respectively. The data in *A* and *B* were fit to a mixed inhibitor model (*solid lines*) and a competitive model (*dashed lines*). In *A* both models fit equally well, leading to the parsimonious conclusion that CO binds competitively. In *B*, a mixed inhibition model fits the data better. Panels *C* and *D* represent Lineweaver–Burke transformations of the data in *A* and *B*, respectively. The data for 0.02 atm CO in *C* is omitted since it would dwarf the rest of the points and the data for 0.0004 atm CO is omitted in *D* since it would overlay with the no CO trace. Two independent replicates of these experiments are shown in the Supporting information.

The inhibition kinetics of CO are shown in Figure 5 and Fig. S13 and summarized in Table 1. There are clear differences in inhibition depending on whether CowN is present or not. We used nonlinear curve fitting to obtain *K*_Ia_ and *K*_Ib_ for CO in the presence and absence of CowN (Fig. 5 and Fig. S13) using a mixed inhibitor model (Equation 2).

In the absence of CowN, *K*_Ia_ is 1.4 × 10^−4^ atm. We were unable to precisely determine *K*_Ib_ since *K*_Ib_ is about 60-fold larger than *K*_Ia_. In such a situation, inhibitor binding to the ES complex is weak and inhibition approaches the competitive case (*K*_Ia_ << *K*_Ib_). This interpretation of the data is supported by Lineweaver–Burke analysis (Fig. 5*C*), which suggests that in the absence of CowN, CO inhibition of *G. diazotrophicus* nitrogenase is well modeled as being competitive with a single *K*_I_ of 1.1 × 10^−4^ atm.

The reason for the large value of *K*_Ib_ in *G. diazotrophicus* (*Gd*) nitrogenase compared with *A. vinelandii* (*Av*) nitrogenase is likely due to small structural differences between the *Gd*- and *Av*-proteins that result in dissimilar CO migration pathways. In addition to the aforementioned Igarashi pathway that reaches FeMoco *via Av*-αG69 and *Av*-αV70 (5, 48), a CO-occupied channel was discovered by crystallography in *Av*-MoFeP (47), suggesting CO may reach FeMoco using two pathways in *Av*-MoFeP. CO in the second channel is surrounded by *Av*-αAla94 and *Av*-βPhe450. In *Gd*-MoFeP, this channel is more polar as it lined by *Gd*-αSer110 and *Gd*-βTyr445 (Fig. S14). Competitive inhibition kinetics in *Gd*-MoFeP agree with a mechanism in which CO has difficulty passing through the Tyr/Ser-lined channel and is mostly limited to reaching FeMoco using the Igarashi channel.

CowN shifts *K*_Ia_ tenfold to 1.43 × 10^−3^ atm in the mixed inhibitor model and *K*_Ib_ is increased about twofold. Using a competitive model, the *K*_I_ increases about fivefold to 6.2 × 10^−4^ atm (Fig. 5, Fig. S13 and Table 1). This indicates CO is a much weaker inhibitor when CowN is present. The fitting results further suggest CowN alters the inhibition mechanism. In the presence of CowN, the data is better represented by a mixed inhibition model since the magnitudes of *K*_Ia_ and *K*_Ib_ are now closer to each other. The mixed inhibition model also provides the superior nonlinear fit. The presence of CowN increases *K*_Ia_. In contrast, the change in *K*_Ib_ is small and the values of *K*_Ib_ with and without CowN are similar to each other. These observations suggest that CowN decreases CO binding/migration to the high-affinity inhibition site (*i.e.*, increases *K*_Ia_) but does not influence the lower-affinity inhibition site as much.

Overall, these data have revealed the following: 1) *G. diazotrophicus* nitrogenase binds C_2_H_2_ as tightly as *A. vinelandii* and CO inhibits *G. diazotrophicus* nitrogenase approximately as tightly as it does *A. vinelandii* nitrogenase, but does so through a competitive mechanism, 2) CowN weakens CO binding between five and tenfold, and 3) CowN primarily relieves CO inhibition to the high-affinity CO-binding site and/or prevents CO migration through the preferred CO access channel.

### CowN protective effect is dependent on both CowN and CO concentration

To test the dose response of CowN protection, we measured nitrogenase activity under 0.1 atm and 0.001 atm CO with increasing concentrations of CowN.

**Figure 6:**
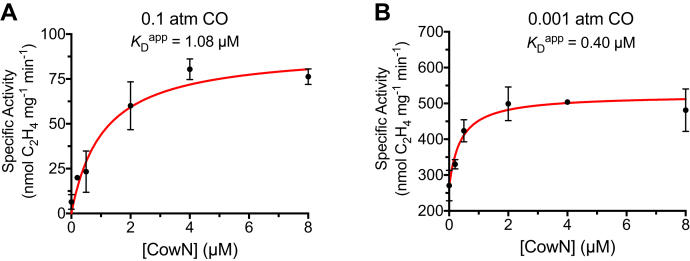
**CowN protection is dose-dependent.***A*, change in nitrogenase activity as a function of CowN under 0.1 atm CO and *B*, 0.001 atm CO. Data in both graphs were fit to a hyperbolic binding equation. Error bars smaller than the size of a datapoint are omitted. Experiments represent a minimum of three replicates.

As shown in Figure 6, nitrogenase activity is hyperbolically dependent on CowN concentration, plateauing at 4 μM CowN with a *K*_D_^app^ equal to 1.08 ± 0.27 μM under 0.1 atm CO and plateauing around 2 μM CowN with a *K*_D_^app^ equal to 0.40 ± 0.15 μM under 0.001 atm CO. The hyperbolic shape of the dose response provides strong evidence that CowN and nitrogenase interact through a specific rather than a nonspecific mechanism, as the latter typically displays linear kinetics.

Unlike under 0.001 atm CO, CowN only partially restores nitrogenase activity under 0.1 atm (Fig. 6), even when CowN is present in high concentrations. The different results for CowN protection under 0.1 and 0.001 atm CO, in terms of both maximum activity and difference in *K*_D_^app^, can be interpreted based on how CO diffuses through nitrogenase to reach FeMoco. As mentioned previously, CO has more than one access pathway to FeMoco and several binding modes (5, 48, 49). The CowN dose response is consistent with a model in which CowN protects nitrogenase efficiently from low concentrations of CO by blocking the preferred CO access channel and/or altering the higher-affinity but not lower-affinity CO-binding sites.

We note, however, that alternative explanations cannot be ruled out to explain the dose–response curves. CowN may bind tighter to uninhibited nitrogenase. Thus, CowN’s binding would appear weaker at high CO concentrations when more nitrogenase is inhibited.

### CowN binds to MoFeP

Enzyme kinetics experiments suggest CowN and nitrogenase interact. We initially attempted to capture CowN–nitrogenase interaction using two methods: EDC cross-linking, which reacts specifically between carboxylic acids and amines (Fig. S15), and pull-down experiments (Fig. S16) under nonturnover and turnover conditions with CO. However, no interaction was detected using these methods, suggesting CowN and nitrogenase may not interact *via* closely paired Glu/Lys salt bridges and that the interaction is likely weak.

Since EDC cross-linking and pull-down experiments did not provide evidence for CowN–nitrogenase complex formation, we turned to a light-activated diazirine-based cross-linker with a spacer length of 8 Å. On one end, the cross-linker attaches nonspecifically to Lys residues *via* N-hydroxysuccinamide (NHS)-ester chemistry. The other end contains a diazirine group that covalently cross-links under UV light irradiation to any residue that is within range. After labeling MoFeP, we confirmed the cross-linker was present by mass spectrometry, which showed covalent attachment to both the α- and β-chain. We also verified that labeling did not damage the MoFeP metal clusters by inductively coupled plasma–optical emission spectroscopy (ICP-OES), which yielded the same Fe:Mo ratios for labeled and unlabeled protein.

**Figure 7:**
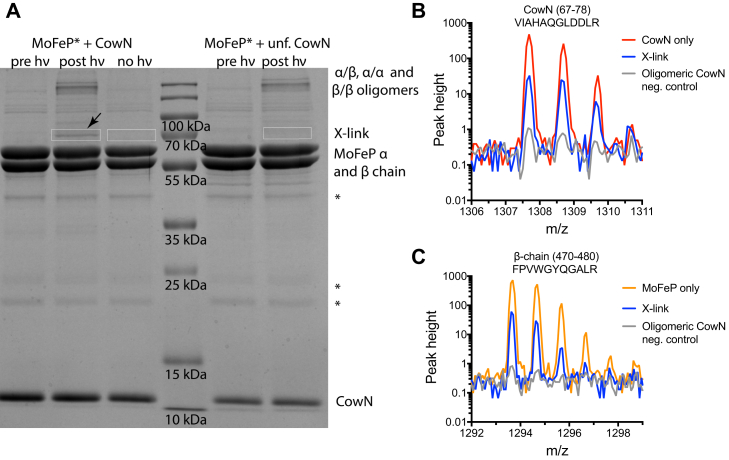
**Cross-linking of MoFeP and CowN.***A*, SDS-PAGE of the cross-linking products between MoFeP^∗^ and CowN. The 70 kDa putative CowN-MoFeP cross-linked pair is marked with an *arrow*. The *boxes* represent the approximate regions of the gel that were excised for tryptic digest and MALDI-TOF MS analysis. Pre hν refers to samples at the start of the cross-linking reaction, post hν refers to samples that were illuminated for 30 min, whereas no hν represents identical samples that were incubated for 30 min without light. Bands marked by an *asterisk* represent impurities in MoFeP. The full gel in panel *A* can be found in the Supporting information. *B*, comparison of the 1307.69 m/z peak from the MoFeP^∗^-CowN cross-linking sample, a CowN-only sample, and the MoFeP^∗^-unfolded CowN negative control. *C*, same as in *B*, but for the 1293.67 m/z peak characteristic of MoFeP β-chain.

Cross-linking experiments were performed separately with diazirine labeled MoFeP (MoFeP^∗^) and labeled CowN (CowN^∗^). The reaction products were resolved by SDS-PAGE. After performing cross-linking experiments with MoFeP^∗^ and CowN, we discovered that a new band reproducibly appears at approximately 70 kDa (Fig. 7*A* and Fig. S17). This band is consistent with the expected molecular weight of either a CowN-MoFeP α-chain or a CowN-MoFeP β-chain cross-linked pair. Several control experiments support the conclusion that this band represents a covalent cross-link between CowN and MoFeP^∗^. The 70 kDa band is not present unless samples are UV-irradiated (Fig. 7*A*) nor is it found in samples containing only MoFeP^∗^ (Fig. S17), and the band’s intensity is dependent on the concentration of CowN (Fig. S18). The intensity of the 70 kDa is very weak when the experiment is conducted in the presence of high salt concentrations, which disrupt protein–protein interactions (Fig. S19). This indicates the band represents an intermolecular complex such as CowN-MoFeP^∗^. Furthermore, there is no cross-linking in the absence of label (Fig. S19). Most importantly, otherwise identical reactions between MoFeP^∗^ and aggregated (misfolded) CowN do not yield a band at 70 kDa, suggesting formation of the 70 kDa band requires folded CowN (Fig. 7*A* and Fig. S17).

To unambiguously confirm that the band at 70 kDa represents a cross-linked MoFeP-CowN pair, we cut out the band from the gel, subjected it to a tryptic digest, and identified the resulting peptides by MALDI-TOF mass spectrometry. If the putative cross-link is in fact between MoFeP and CowN, we expected to find peptides belonging to both proteins in the excised band.

Mass spectroscopy revealed that peptides attributed to both CowN and MoFeP were present in the 70 kDa band. Notably, a prominent CowN peak at 1307.69 m/z and a characteristic MoFeP β-chain peak with m/z = 1293.67 were discovered in the digested 70 kDa cross-link band (Fig. 7, *B* and *C*). A full list of peptides found in these experiments is presented in the Supporting information.

The sequence coverage for CowN and MoFeP is shown in Table S1. We were able to map 20% of CowN and 19% and 22% of MoFeP’s α-chain and β-chain, respectively. Furthermore, fragment ion analysis on the characteristic CowN peak at 1307.69 m/z confirmed that this peptide belongs to CowN (Table S2). Together, these data indicate that the 70 kDa band is a CowN-MoFeP cross-linked pair and thus that CowN and MoFeP interact.

Control experiments lend further confidence to our interpretation of the results. When we excised the 70 kDa region from gel lanes from reactions containing only MoFeP^∗^, MoFeP^∗^ and aggregated CowN, or MoFeP^∗^ and CowN that were kept in the dark, neither MoFeP nor CowN peptides were detected by MALDI-TOF MS (Fig. 7 and Fig. S20).

Based on the presence of both MoFeP α-chain and MoFeP β-chain peptides in the 70 kDa cross-linking band, it is likely that CowN interacts with both MoFeP chains. While our cross-linking experiments demonstrate that CowN and MoFeP interact, we were not able to determine the interaction site using either MALDI-TOF mass spectrometry or, in a separate experiment, LC mass spectrometry–based methods. The inability to find the cross-linking site is likely due to the nonspecific nature of the diazirine labeling reaction. There are multiple Lys residues on the MoFeP surface. CowN probably interacts with several Lys residues leading to a low cross-linking abundance for a particular CowN-MoFeP fragment.

Cross-linking experiments between diazirine labeled CowN (CowN∗) and unlabeled MoFeP did not yield cross-linked pairs since CowN∗ degraded under UV illumination. We also investigated CowN cross-linking with MoFeP∗ in the presence of FeP under turnover conditions; however, no cross-linking was detected under these conditions. This may indicate that FeP and CowN compete for a similar location on the MoFeP surface.

### CowN does not alter the resting electronic state of nitrogenase

**Figure 8:**
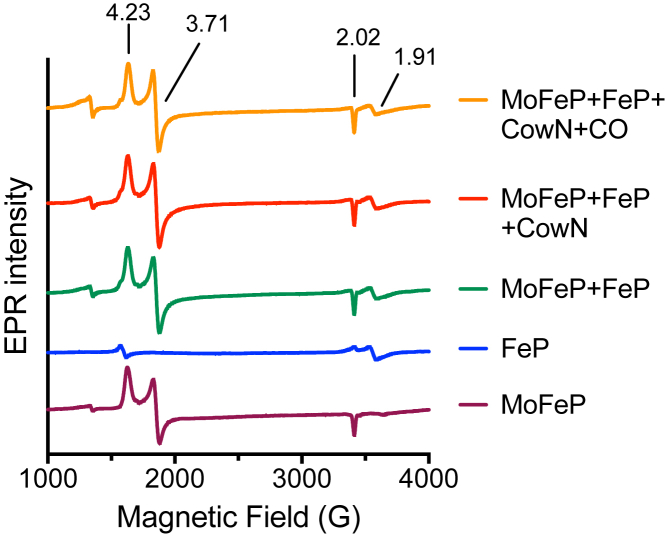
**EPR spectra of FeP and MoFeP under N**_**2**_**and, where indicated, CO.** Spectra are identical in the presence and absence of CowN, suggesting CowN does not alter the electronic state of nitrogenase under the experimental conditions. Values above the spectra represent the *g*-factors.

The data, so far, are consistent with a mechanism in which CowN interacts with nitrogenase and selectively allows nitrogenase to reduce substrate while weakening CO inhibition. We hypothesize that CowN may interact with MoFeP in vicinity of FeMoco. Such an interaction would enable CowN to alter gas access or elicit changes to FeMoco’s local environment to disfavor CO binding. We examined nitrogenase (FeP and MoFeP together) by X-band EPR spectroscopy under nonturnover conditions (no ATP present) under N_2_ with and without CO. Under these experimental conditions FeP and MoFeP likely interact in an “encounter complex” (50, 51). We were expecting to detect changes in FeMoco’s S = 3/2 spin state if CowN interaction occurred near FeMoco. Other potential interactions, such as CowN binding near FeP’s metal cluster, could also be detected in this assay. However, all samples displayed the characteristic S = 3/2 signal of MoFeP and S = 1/2 signal of FeP and were identical to each other (Fig. 8 and Fig. S9). This indicates that CowN either does not alter nitrogenase’s electronic structure or that CowN does not bind nitrogenase under nonturnover conditions in the vicinity of any of its metal clusters. Results from similar experiments under turnover conditions were inconclusive since little activity, as inferred through EPR signal changes, was seen.

## Conclusions

We have purified *G. diazotrophicus* CowN and shown that it acts directly on nitrogenase to protect it from the inhibitor CO. This establishes CowN as an important auxiliary protein that helps support bacterial nitrogen fixation. As such, CowN joins a growing list of proteins that includes Shethna protein (27), DraT/DraG (52) and terminal oxidases (53), whose roles are to protect nitrogenase from inhibitors. CowN is unique among nitrogen fixation proteins since it is the only one known to interact with nitrogenase during turnover other than electron-donating flavodoxins (54) and ferredoxins (55). CowN is the first known protein that can modulate nitrogenase reactivity, suggesting that nitrogenase chemistry may be influenced by protein–protein interactions to a greater extent than is currently assumed.

Our data indicate CowN operates by allowing nitrogenase to maintain substrate reduction activity in the presence CO, ruling out alternative protective mechanisms. Although CowN does not fully protect nitrogenase at high CO concentrations, CowN supports nearly full nitrogenase activity at lower CO levels (<0.001 atm) likely encountered by diazotrophs in the environment (56). Our observations explain earlier results that diazotrophs are able to grow under nitrogen fixing conditions in the presence of CO (20). The *K*_I_ of CO in the presence of CowN is very similar to the one reported for CO inhibition of nitrogenase *in vivo* (∼0.002 atm) (11), suggesting that the higher tolerance of nitrogenase *in vivo* compared with purified nitrogenase is due to CowN activity.

**Figure 9:**
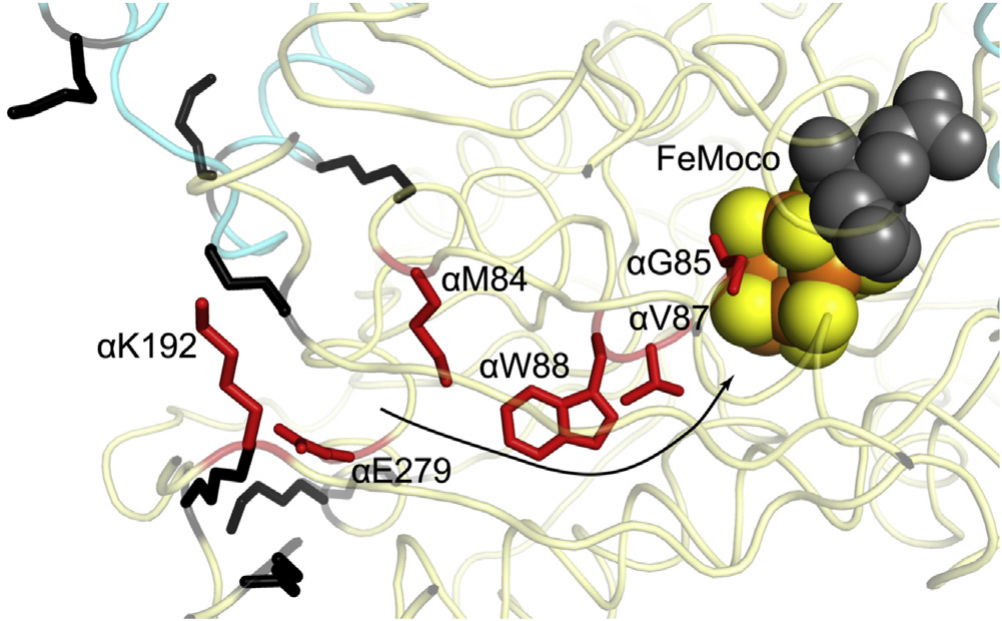
**Possible CowN-binding site near a proposed hydrophobic CO access channel in *Gd*-MoFeP (PDB: 5KOH).** Key residues that line the entrance and interior of the channel are highlighted in *red* and annotated using *G. diazotrophicus* nitrogenase numbering. The *arrow* represents the proposed CO migration route. Lys residues that surround the channel entrance and may mediate interaction with CowN are highlighted in *black*. The MoFeP α-chain is colored in *light yellow* and the β-chain is colored *cyan*.

Based on the inability of CowN to pull down MoFeP, it is unlikely that CowN forms a tight complex with MoFeP. Furthermore, CowN binding is likely different compared with δ-subunit binding to VFeP based on the lack of change of EPR signal in the presence of CowN. Although we were unable to locate the CowN-MoFeP binding site, we hypothesize that CowN interacts with MoFeP near the opening of the Igarashi channel (Fig. 9). The entrance to this channel lies at interface of the α- and β-chain of MoFeP and is surrounded by several Lys residues. CowN binding to this location would be consistent with our observation that CowN cross-links to both MoFeP chains. Furthermore, the presence of Lys residues near the channel opening provides a charge-complementary surface for negatively charged CowN (Fig. 1 and Fig. S1). Finally, the potential binding site is located far away from either FeMoco or P-cluster, in line with our EPR results that showed CowN does not influence MoFeP’s electronic state under resting conditions.

Future research will be directed at revealing where CowN and MoFeP interact and examining how CowN alters CO migration and binding to FeMoco. Furthermore, the observation that CowN is expressed under all nitrogen fixation conditions merits further study since it suggests CowN may have additional roles in nitrogen fixation beyond CO protection.

## Experimental procedures

### Reagents

All reagents were purchased from Thermo-Fisher, VWR, or Sigma Aldrich and were ACS grade or equivalent. Gases were obtained from Westair unless otherwise specified.

### Nitrogenase expression and purification

*G. diazotrophicus* was grown in LGI media (30) containing 5 mM potassium phosphate, pH 6, and 0.5 mM (NH_4_)_2_SO_4_. Cell were grown in 6 L Erlenmeyer flasks that were filled to 2.5 L. Growth was started by adding 20 mL of a starter culture (OD_600nm_ ∼ 1) to each flask, and cultures were grown at 30 °C with a shaker speed of 200 rpm. After about 4 to 5 days, the optical density reached 0.6 to 0.8, at which point nitrogenase in *G. diazotrophicus* became derepressed and nitrogenase activity could be observed using acetylene reduction assays, as described previously (30). Cells were harvested at an OD_600nm_ ∼1.0 to 1.4 by centrifugation at 5000 rpm and stored at −80 °C until use. Cells were lysed by microfluidization at 16,000 to 18,000 psi. Sodium dithionite (DT) was added to the lysate to a final concentration of 5 mM, the lysate was degassed under Ar and spun down in airtight centrifuge bottles for 45 min at 13,000 rpm. At this point, all protein manipulation was conducted under an Ar atmosphere using degassed buffers. The lysate supernatant was loaded onto a DEAE column that had been pre-equilibrated with a buffered solution containing 50 mM Tris, pH 8, 100 mM NaCl, 5 mM DT. Nitrogenase was eluted using a linear salt gradient with 50 mM Tris, pH 8, 500 mM NaCl, 5 mM DT. Fractions containing FeP and MoFeP were detected by SDS-PAGE. FeP and MoFeP were concentrated using an Amicon stirred cell to about 5 to 10 mL. FeP and MoFeP were further purified on an S200 gel filtration column pre-equilibrated with a buffered solution containing 50 mM Tris, pH 8, 500 mM NaCl, 5 mM DT. Protein purity was determined by SDS-PAGE, and the integrity of metal clusters on MoFeP verified by ICP-OES. FeP and MoFeP were concentrated and stored under liquid nitrogen until use.

### Molecular cloning of *cowN*

Genomic DNA was extracted from *G. diazotrophicus* using a GeneJet genomic extraction kit (Thermo-Fisher) to use for cloning *cowN* (Gdia_2893). His-CowN (henceforth referred to as CowN) was amplified using following primers by PCR.

Forward: 5’ CGC CAT ATG ACC GAG CAG ATC GAC CG

Reverse: 5’ GGC GAG CTC TTA CTA CAT GCA CAG GAC TTC G

The forward and reverse primers have an NdeI and SacI restriction enzyme site, respectively, and were inserted into pET28a (Millepore-Sigma) by restriction digest cloning.

To generate MBP-CowN, CowN was amplified using following primers:

Forward: 5’ CGC GAG CTC ATG ACC GAG CAG ATC G

Reverse: 5’ GGC GGA TCC TTA CTA CAT GCA CAG GAC TTC G

The forward and reverse primers have a SacI and BamHI restriction enzyme site, respectively, used for cloning CowN into pMAL-c5x (New England Biolabs) using restriction digest methods. The DNA sequences for both CowN constructs were verified by DNA sequencing (Genscript).

### CowN expression and purification

CowN was expressed in *E. coli* (BL21) in LB (Miller) broth. Expression was induced by addition of IPTG to a final concentration of 400 μM when cells reached an OD_600nm_ between 0.7 and 0.9. Cells were lysed by sonication and spun down at 12,500 rpm for 1 h. CowN, which is located in the pellet, was resuspended in a buffered solution containing 50 mM Tris, pH 8, 500 mM NaCl, 6 M Urea and gently shaken overnight. The solution containing CowN was then spun down for 20 min at 5000 rpm to remove undissolved protein, and the supernatant was and loaded onto a Ni^2+^ HiTrap column (GE healthcare) equilibrated with 50 mM Tris, pH 8, 500 mM NaCl, 6 M Urea, 20 mM imidazole. CowN was eluted using a linear gradient with a solution containing 50 mM Tris, pH 8, 500 mM NaCl, 500 mM imidazole, and 6 M urea. Fractions containing CowN were identified by SDS-PAGE and pooled. EDTA was added to CowN to a concentration of 10 mM. To refold CowN, the protein was dialyzed stepwise against following buffered solutions: 1) 50 mM Tris, pH 8, 200 mM NaCl, 10 mM EDTA, 2 M urea; 2) 50 mM Tris, pH 8, 200 mM NaCl; 3) 50 mM Tris, pH 8, 100 mM NaCl. Each dialysis step proceeded for at least 6 h. After dialysis, CowN was concentrated using an Amicon centrifugal concentrator and spun down at 12,500 rpm to remove precipitated protein. CowN was then loaded onto an S75 gel filtration column (GE healthcare), equilibrated with a solution containing 25 mM HEPES, pH 8, 25 mM NaCl, to separate monomeric CowN from soluble CowN aggregate.

### MBP-CowN expression and purification

MBP-CowN was expressed using the same procedure as CowN. Cells were lysed by sonication, spun down for 1 h at 12,500 rpm, and the supernatant loaded onto an an MBP-TRAP column (GE Healthcare), equilibrated with a solution containing 20 mM Tris, pH 7.4, 200 mM NaCl, 1 mM EDTA. MBP-CowN was eluted using a linear gradient containing 20 mM Tris, pH 8, 200 mM NaCl, 1 mM EDTA, 10 mM maltose. MBP-CowN was further purified on an S200 gel filtration column equilibrated with 25 mM Tris, pH 8, 100 mM NaCl, and protein purity was verified by SDS-PAGE.

### Real-time quantitative PCR experiments

*G. diazotrophicus* for RT-qPCR experiments were grown in LGI media, as described above. In ammonia-replete N^+^ cultures, the concentration of (NH_4_)_2_SO_4_ was equal to 10 mM (*versus* 0.5 mM in normal N^−^ media). Experiments to test the effect of CO on CowN expression began when cells reached mid-to-late exponential phase (OD between 0.6 and 0.8), at which point nitrogenase repression is turned off in N^−^ cultures. Nitrogenase activity in N^−^ cultures was verified by monitoring C_2_H_2_ reduction. No nitrogenase activity, as measured using C_2_H_2_ reduction, was detected in N^+^ media samples. Cells (3 mL) were placed in 10 mL stoppered vials and CO was added to a partial pressure of 0.05 atm (5%) using an airtight Hamilton syringe. After 1 h, the stopper was removed and cells were allowed to oxygenate for ∼5 min. The vials were stoppered again, CO added to 0.05 atm, and cells incubated for another 70 min. The rationale for the initial CO induction is to give the bacteria sufficient time to mount a CO response. After the second induction, cells were rapidly harvested by centrifugation and stored at −80 °C until use. Cultures that were not exposed to CO were prepared in an identical manner, except that no CO was added to the stoppered vials.

Messenger RNA was harvested using a PureLink RNA extraction kit (Thermo-Fisher) and mRNA integrity verified by denaturing agarose gel electrophoresis. RT-qPCR experiments were conducted using a Luna one-step RT-qPCR kit (New England Biolabs) using 200 ng of RNA with primers listed in Table S3. Expression was normalized to obtain ΔC_t_ values using the housekeeping gene *rpoD*, which has been validated as a RT-qPCR control in *G. diazotrophicus* (57). To determine ΔΔC_t_ values, expression of *nifK* and *cowN* was compared pairwise between cultures grown in N^−^ and N^+^ media without CO, between cultures grown in N^−^ media with and without CO, and between cultures grown in N^+^ media with and without CO.

### Circular dichroism spectroscopy

Circular dichroism spectra of CowN were taken on a Jasco J-1500 CD spectrophotometer. CowN, typically at a concentration of 0.2 mg/ml, was in a buffered solution containing 5 mM HEPES, pH 8, 5 mM NaCl. Spectra were recorded at 4 °C using scan rate of 100 nm/min, a data pitch of 0.2 nm, 1.0 nm bandwidth, and 2 s integration time. A total of five spectra were averaged per experiment. For thermal denaturation assays, the CD signal at 222 nm was recorded as the temperature was slowly increased to 94 °C. An integration time of 2 s and bandwidth of 1 nm were selected for these experiments. Five acquisitions were averaged per experiment. To calculate percent unfolded protein, the CD signal (in mdeg) was converted such that signal at 4 °C is equal to 0% unfolded and the signal at 94 °C is equal to 100% unfolded. The *T*_m_ was determined by finding the peak of the first derivative of the denaturation curve using Origin Pro (58).

### Dynamic light scattering

Dynamic light scattering experiments were carried out in a Wyatt DynaPro Nanostar instrument at room temperature. The instrument wavelength was equal to 532 nm and the detector angle was 163.5°. Each measurement consisted of a minimum of 20 acquisitions, with an acquisition time set to 5 s. The CowN concentration is indicated in the main text, and the buffered solution used in DLS experiments contained 25 mM HEPES, pH 8, 25 mM NaCl.

### ICP-OES procedures

MoFeP was diluted to 0.05 to 0.5 mg/mL in a solution containing 6 M guanidine hydrochloride to denature the protein and 2% nitric acid to liberate the metal ions and precipitate the polypeptide. The solution was clarified by centrifugation. Metal content of the respective proteins was measured on an Agilent 5110 ICP-OES at 238.2 nm for Fe and 203.8 nm for Mo. The metal content was calculated based on standard curves for Fe and Mo that spanned 0 to 500 ppm.

### Enzymatic assays

Nitrogenase assays were conducted under an atmosphere of Ar in 1.135 mL of buffered solution containing 50 mM Tris, pH 8, 60 mM NaCl, 5 mM ATP, 5 mM MgCl_2_, 5 mM DT, and 10 mg/mL creatine phosphate and 0.125 mg/mL creatine kinase, which serve to maintain a constant ATP concentration, in stoppered 10 mL vials. Gaseous C_2_H_2_ and CO were filled into evacuated round-bottom flasks and vented to 1 atm. The respective gases were then added to the stoppered vials using airtight Hamilton syringes to the pressures indicated in the main text. Unless otherwise noted, protein concentrations were 0.2 μM, 2 μM, and 2 μM for MoFeP, FeP, and CowN, respectively. For experiments lacking a protein component, the concentrations were the same, unless specified otherwise. Assays were initiated by addition of FeP and proceeded for 20 min at 30 °C before being terminated by addition of 0.3 mL of 4 M NaCl, which stops nitrogenase activity (59). Gaseous reaction products were measured by on-column injection of 0.2 mL of headspace onto an SRI 310 gas chromatograph fitted with a Hyesep Q column. Hydrocarbon gases were detected using a flame ionization detector and, where indicated, CO was detected using a methanizer. A C_2_H_4_ standard curve (Mesa Gas) was constructed to convert C_2_H_4_ peak areas to molar quantities. All assays were repeated, at minimum, three times. In CowN dose-dependence experiments, data was fit to a hyperbolic binding curve, $b=\frac{B_{max}\left[CowN\right]}{K_{D}^{app}+\left[CowN\right]}$, where *B*_max_ is the maximum amount of binding and *K*_D_^app^ is the binding constant for CowN at a given CO concentration. For *K*_M_ and *K*_I_ determination, data was fit to Equation 2 using nonlinear curve fitting. A two-tailed *t*-test was used to determine the statistical significance of the differences in enzyme activity. All curve fitting and statistical analysis were done in Graphpad Prism.

### Labeling MoFeP and CowN with a diazirine-based cross-linker

MoFeP was labeled at room temperature at a concentration of 15 mg/mL with a tenfold molar excess of NHS-diazirine (sulfosuccinimidyl 6-(4,4′-azipentanamido)hexanoate, Thermo Fisher) in a buffered solution containing 50 mM Hepes, pH 8, and 250 mM NaCl. CowN was labeled at room temperature at a concentration of approximately 1.3 mg/mL with 40-fold molar excess NHS-diazirine in a buffered solution of 50 mM Hepes, pH 8, and 25 mM NaCl. For both proteins the labeling reaction was terminated after 30 min by addition of Tris, pH 8, to final concentration of 100 mM. Excess label was then removed on a desalting column. The presence of the cross-linker on MoFeP was confirmed by mass spectrometry. The integrity of the metal clusters on MoFeP after adding the cross-linker was confirmed by ICP-OES.

### Cross-linking assays

Cross-linking assays with both labeled CowN and labeled MoFeP were performed in a buffered solution containing 50 mM Tris, pH 8, and 60 mM NaCl. The protein concentrations were 1.25 μM MoFeP and 15 μM CowN, unless specified otherwise. Cross-linking was achieved by illuminating samples in unstoppered conical vials in an Ar-filled glovebag for 30 min with a 365 nm handheld 8 W UV-lamp. For assays conducted under turnover conditions, the concentrations of CowN, MoFeP^∗^, and FeP were 2 μM, 0.2 μM, and 2 μM respectively, and assays were carried out in a buffered solution containing 50 mM Tris, pH 8, and 60 mM NaCl and 5 mM ATP in sealed UV-cuvettes. Cross-linking products were resolved by 10% SDS-PAGE and proteins stained using Coomassie.

### Tryptic digest of cross-linking products and mass spectrometry

Bands corresponding to MoFeP, CowN, or the putative cross-linked pair were excised from a polyacrylamide gel using a clean razor blade. Samples were prepared for tryptic digest by destaining the respective bands in a 1:1 mixture of 200 mM ammonium bicarbonate and acetonitrile, reducing the proteins with DTT for ∼10 min at 80 °C, and then alkylating Cys residues with iodoacetamide. The gel bands were then washed with a 200 mM ammonium bicarbonate solution followed by acetonitrile and then dried. The bands were then rehydrated in the 200 mM ammonium bicarbonate solution and the proteins digested with 100 to 250 ng trypsin for 18 h at 37 °C. The content of the respective bands was then extracted with a small volume (∼20 μl) of 1% formic acid. To prepare the spot, 1 μl of extracted protein was mixed with a saturated α-Cyano-4-hydroxycinnamic acid (CHCA) solution in 1:1 0.1% trifluoroacetic acid and allowed to dry at room temperature. The dried spots were analyzed by MALDI-TOF/TOF in positive reflector mode on an AB SCIEX 5800. A total of 6000 laser shots were accumulated into an average spectrum. The data was peak picked and mapped to an *in silico* digest of the corresponding protein sequences using mMass.org open-source software. TOF/TOF collision-induced dissociation (CID) fragmentation was used to confirm b&y ions from the CowN peptide fragment, 1307.69 m/z, in both the CowN and cross-linked samples.

### EDC cross-linking

EDC cross-linking was conduced in a buffered solution containing 50 mM HEPES, pH 8, 60 mM NaCl, 5 mM DT. For experiments that were done under turnover conditions, reactions also contained 5 mM MgCl_2_ and 5 mM ATP. For experiments with CO, the reaction was carried out in stoppered vials and CO was added *via* a gastight syringe to a final concentration of 0.1 atm. The protein concentrations were 2.5 μM MoFeP, 15 μM FeP, and when present, 15 μM CowN. Cross-linking was initiated by addition of EDC to a final concentration of 12.5 mM. Reactions proceeded for 30 min and were stopped by transferring 10 μl reaction aliquots into 90 μl of 200 mM sodium acetate solution. Proteins were resolved on a 10% SDS-PAGE.

### Pull-down experiments

Pull-down experiments were conducted using the same parameters as EDC cross-linking experiments with the expectation that 50 mM Tris, pH 8 was used instead of HEPES and the DT concentration was 1 mM to prevent stripping of the Ni-NTA resin. Proteins were pulled down after a 10 min incubation with 50 μl of Ni-NTA resin (Thermo-Fisher) and subjected to three wash steps to remove weakly and nonspecifically bound protein from the beads. The wash buffers contained 1) 50 mM Tris, pH 8, 60 mM NaCl, 5 mM DT, 2) 50 mM Tris, pH 8, 60 mM NaCl, 5 mM DT,10, mM imidazole, 3) 50 mM Tris, pH 8, 60 mM NaCl, 5 mM DT, 500 mM Imidazole. Proteins were resolved by a 10% SDS-PAGE. Control experiments demonstrated that CowN, on its own, is efficiently pulled down using this experimental setup.

### EPR experiments

EPR experiments were carried out with 25 μM FeP and 25 μM MoFeP. When present, the CowN concentration was 50 μM. Proteins were prepared under an N_2_ atmosphere in a solution containing 50 mM Tris, pH 8, 60 mM NaCl, 10 mM DT. Where indicated, CO was added to a partial pressure of 0.1 atm. Spectra were taken on a Bruker EMX spectrometer equipped with an ER041XG microwave bridge, an Oxford Instrument liquid He quartz ESR 900 cryostat, and a dual-mode cavity (ER4116DM) cryostat at 10K. Each spectrum represents the average of four scans. Instrument settings were: Instrument power, 6.4 mW; attenuation, 15 dB; modulation amplitude, 10.2 G; conversion time, 40.96 ms; time constant, 0.01 ms; gain, 30 dB; frequency, 9.64 GHz.

## Data availability

All data is contained in the article and supporting information.

## Supporting information

This article contains supporting information (30, 61).

## Conflict of interest

The authors declare no conflict of interest with the content of the article.
