# Supplementary material for: CowN sustains nitrogenase turnover in the presence of the inhibitor carbon monoxide
Source: J Biol Chem. 2021 Mar 2;296:100501. doi: 10.1016/j.jbc.2021.100501 (PMC8047169; doi:10.1016/j.jbc.2021.100501)
Supplement: Supplemental Figures S1–S20 and Tables S1–S3 [file mmc1.pdf]

Supporting information for:

CowN sustains nitrogenase turnover in the presence of the inhibitor carbon monoxide

Michael S. Medina,[1] Kevin O. Bretzing,[1] Richard A. Aviles,[1] Kiersten M. Chong,[1] Alejandro Espinoza,[1] Chloe Nicole G. Garcia,[1] Benjamin B. Katz,[2] Ruchita N. Kharwa,[1] Andrea Hernandez,[1] Justin L. Lee,[2] Terrence M. Lee,[1] Christine Lo Verde,[1] Max W. Strul,[1] Emily Y. Wong,[1] Cedric P. Owens[1]*

[1] Schmid College of Science and Technology, Chapman University, Orange, CA 92701, United States

[2] Department of Chemistry, University of California, Irvine, Irvine, CA 92697, United States

* Corresponding author: Cedric Owens
Email: cpowens@chapman.edu

Table S1. Sequence coverage of MoFeP and CowN in 70 kDa cross-link band and from purified proteins after SDS-PAGE, as identified by MALDI-TOF mass spectrometry after tryptic digest. Regions of the protein that were identified are highlighted in green.

**Cross-linking product from excised 70 kDa band**
**CowN (20% coverage)**<br>MGSSHHHHHHSSGLVPRGSHMTEQIDRYVSFRNVEWERRTAEVFALLQPHFDGSTSPFWDYFLRQRVIA<br>HAQGLDDLRVLHNFLPTLKDLLEELDDGRTLSRLEELEVLCM
**MoFeP α-chain (19% coverage)**<br>MSLDEDKTNDSAFHARLIAEVLEAYPDKARKRRQKHLNVAGQAEAEAQDAGEEGVMLSECDVKSNVKSV<br>PGVMTIRGCAYAGSKGVVWGPVKDMVHISHGPVGCGQYSWSQRRNYYIGNTGVDSFVTMQFTSDFQEKD<br>IVFGGDKKLEKIIDEIDELFPLAKGISVQSECPIGLIGDDIEAVSRKKKKEIGKTIVPVRCEGFRGVSQ<br>SLGHHIANDAIRDWVFDGEDKHAAFETTPYDVNVIGDYNIGGDAWSSRILLEEMGLRVVGNWSGDATLA<br>EIERAPKAKLNLIHCYRSMNYICRHMEEKYNIPWTEYNFFGPSQIAASLRKIAALFDEKIQEGAERVIA<br>KYQPLVDAVIEKFRPRLAGKKVMLYVGGLRPRHVVNAYNDLGMEIVGTGYEFGHNDDYQRTGHYVREGT<br>LIYDDVTGYELEKFIEGIRPDLVGSGIKEKYPVQKMGIPFRQMHSWDYSGPYHGYDGFAIFARDMDLAI<br>NNPVWSMFKAPWKNAA
**MoFeP β-chain (22% coverage)**<br>MPQNVDKILDHAPLFREPEYQEMLAGKAKLENMPPADKVVEIADWTKSWEYREKNFARESLSVNPAKAC<br>QPLGAVFVASGFERTMSFVHGSQGCVAYYRSHLSRHFKEPSSAVSSSMTEDAAVFGGLNNMVDGLANTY<br>KLYDPKMIAVSTTCMAEVIGDDLHAFIQTAKGKGSVPEEFDVPFAHTPAFVGSHVTGYDNMLKGILEHF<br>WKGRTPVPNRSVNIIPGFDGFAVGNNRELKRILGMMGVQYTILSDVSDQFDTPSDGEYRMYDGGTKIEA<br>ARDAVNADYTISLQEYCTPKTLEYCQSFGQKTASFHYPLGIGATDDLLQKLSEISGKPVPQELEMERGR<br>LVDALADSQAYLHGKTYAIYGDPDFVYGMARFILETGGEPKHCLATNGSKAWEAQMQELFDSSPFGVGC<br>KAWGGKDLWHMRSLLATEKVDLLIGNSYGKYLERDTDTPLIRLMFPIFDRHHHHRFPVWGYQGALRVLV<br>TLLDKIFDKLDDDTIQAGVTDYSFDLTR
**Sequence coverage of purified proteins after SDS-PAGE and tryptic digest.**
**CowN only excised from ~13 kDa band (54% coverage)**<br>MGSSHHHHHHSSGLVPRGSHMTEQIDRYVSFRNVEWERRTAEVFALLQPHFDGSTSPFWDYFLRQRVIA<br>HAQGLDDLRVLHNFLPTLKDLLEELDDGRTLSRLEELEVLCM
**MoFeP only excised from ~55-57 kDa bands**<br><br>**α-chain (32% coverage)**<br>MSLDEDKTNDSAFHARLIAEVLEAYPDKARKRRQKHLNVAGQAEAEAQDAGEEGVMLSECDVKSNVKSV<br>PGVMTIRGCAYAGSKGVVWGPVKDMVHISHGPVGCGQYSWSQRRNYYIGNTGVDSFVTMQFTSDFQEKD<br>IVFGGDKKLEKIIDEIDELFPLAKGISVQSECPIGLIGDDIEAVSRKKKKEIGKTIVPVRCEGFRGVSQ<br>SLGHHIANDAIRDWVFDGEDKHAAFETTPYDVNVIGDYNIGGDAWSSRILLEEMGLRVVGNWSGDATLA<br>EIERAPKAKLNLIHCYRSMNYICRHMEEKYNIPWTEYNFFGPSQIAASLRKIAALFDEKIQEGAERVIA<br>KYQPLVDAVIEKFRPRLAGKKVMLYVGGLRPRHVVNAYNDLGMEIVGTGYEFGHNDDYQRTGHYVREGT

LIYDDVTGYELEKFIEGIRPDLVGSGIKEKYPVQKMGIPFRQMHSWDYSGPYHGYDGFAIFARDMDLAI
NNPVWSMFKAPWKNAA

MoFeP only excised from ~55-57 kDa bands

β-chain (40% coverage)

MPQNVDKILDHAPLFREPEYQEMLAGKAKLENMPPADKVVEIADWTKSWEYREKNFARESLSVNPAKAC
QPLGAVFVASGFERTMSFVHGSQGCVAYYRSHLSRHFKEPSSAVSSSMTEDAAVFGGLNNMVDGLANTY
KLYDPKMIAVSTTCMAEVIGDDLHAFIQTAKGKGSVPEEFDVPFAHTPAFVGSHVTGYDNMLKGILEHF
WKGRTPVPNRSVNIIPGFDGFAVGNNRELKRILGMMGVQYTILSDVSDQFDTPSDGEYRMYDGGTKIEA
ARDAVNADYTISLQEYCTPKTLEYCQSFGQKTASFHYPLGIGATDDLLQKLSEISGKPVPQELEMERGR
LVDALADSQAYLHGKTYAIYGDPDFVYGMARFILETGGEPKHCLATNGSKAWEAQMQELFDSSPFGVGC
KAWGGKDLWHMRSLLATEKVDLLIGNSYGKYLERDTDTPLIRLMFPIFDRHHHHRFPVWGYQGALRVLV
TLLDKIFDKLDDDTIQAGVTDYSFDLTR

Table S2. Fragment ion table for the CowN peptide VIAHAQGLDDLR. Bolded fragment ions denote those that were detected in a TOF/TOF collision-induced dissociation fragmentation experiment. The results confirm that the prominent peak at 1307.69 m/z originates from the VIAHAQGLDDLR peptide. For comparison, the experimentally detected fragment ions from a CowN-only sample are also listed.

	Seq	#	B	Y	# (+1)
Cross-link	V	1	**100.07574**	1307.70667	12
	I	2	213.15980	1208.63825	11
	A	3	284.19691	1095.55419	10
	H	4	**421.25582**	1024.51708	9
	A	5	**492.29294**	887.45816	8
	Q	6	**620.35151**	816.42105	7
	G	7	**677.37298**	688.36247	6
	L	8	**790.45704**	631.34101	5
	D	9	**905.48398**	518.25695	4
	D	10	**1020.51093**	403.23000	3
	L	11	**1133.59499**	**288.20306**	2
	R	12	**1289.69610**	**175.11900**	1
CowN-only	V	1	100.07574	1307.70667	12
	I	2	213.15980	1208.63825	11
	A	3	284.19691	1095.55419	10
	H	4	**421.25582**	1024.51708	9
	A	5	**492.29294**	887.45816	8
	Q	6	**620.35151**	816.42105	7
	G	7	**677.37298**	688.36247	6
	L	8	**790.45704**	631.34101	5
	D	9	**905.48398**	518.25695	4
	D	10	**1020.51093**	403.23000	3
	L	11	**1133.59499**	**288.20306**	2
	R	12	**1289.69610**	**175.11900**	1

Table S3. Primers used for RT-qPCR

**Sequence name**	**Sequence**
cowN forward	5' TTC CGG AAC GTG GAA TGG
cowN reverse	5' CCC GTC ATC CAG CTC TTC
nifK forward	5' GGT ATC CTG GAG CAT TTC TG
nifK reverse	5' CTT CAG CTC GCG ATT GTT
rpoD forward	5' CTG AAG ATC GCC AAG GAA C
rpoD reverse	5' CGT CTT GTC CTC GAT GAA ATC

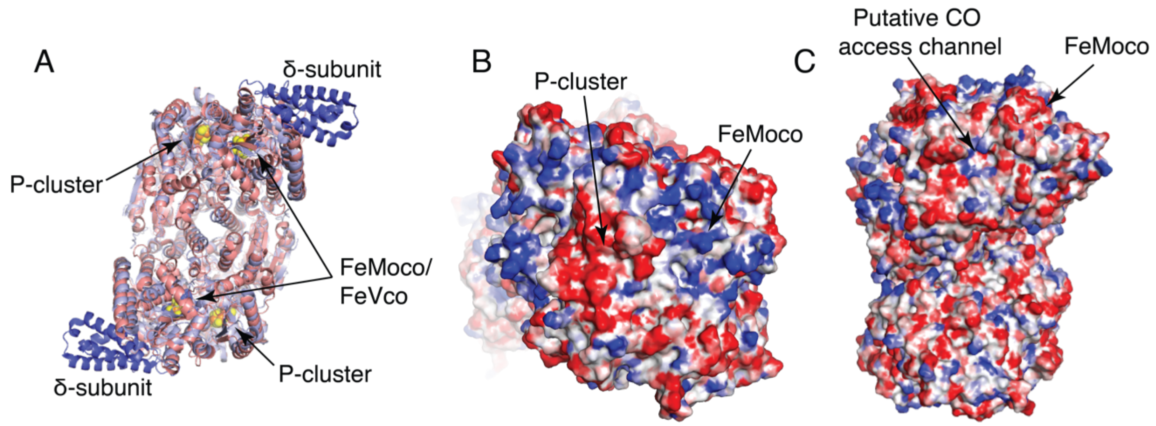

Figure S1. (A) Structural alignment of MoFeP (PDB: 3U7Q) to VFeP (PDB: 5N6Y). MoFeP is colored in pink and VFeP is light blue. The δ-subunit of VFeP is shaded in dark blue. The respective metal clusters are shown as spheres. **(B)** and **(C)** Electrostatic surface map of MoFeP, calculated using the Bluues server (1), where negatively and positively charged regions are colored red and blue, respectively. The location of P-cluster and FeMoco under the protein surface are indicated, as is the location of a possible CO access pathway.

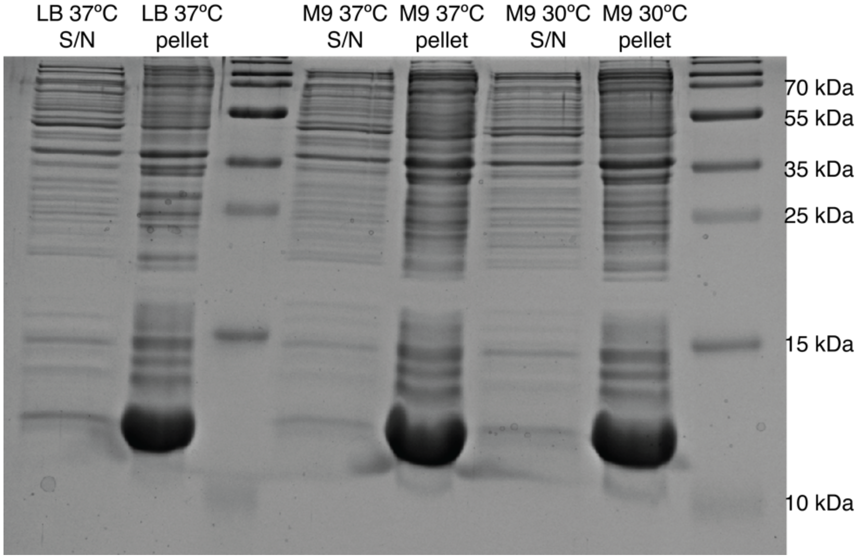

Figure S2. SDS-PAGE of CowN expressed in LB and M9 minimal media at different temperatures demonstrating that the protein is found in inclusion bodies under all tested conditions.

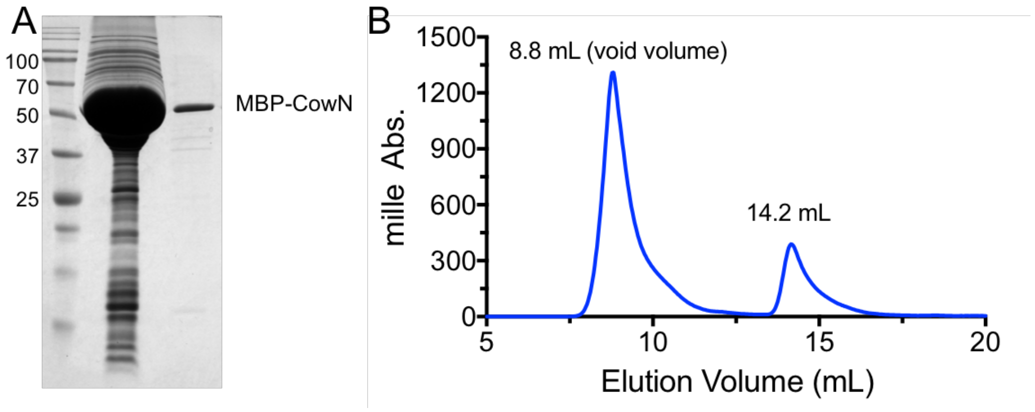

Figure S3. (A) SDS PAGE of purified MBP-CowN at two different concentrations. **(B)** Chromatogram of MBP-tagged CowN run on an S200 gel filtration column showing a large peak at the void volume of 8.8 mL containing aggregated but soluble protein. The 14.2 mL peak is calculated to have the molecular weight of 80.7 kDa, lying between the expected molecular weight of a MBP-CowN monomer (54.1 kDa) and dimer (108.2 kDa).

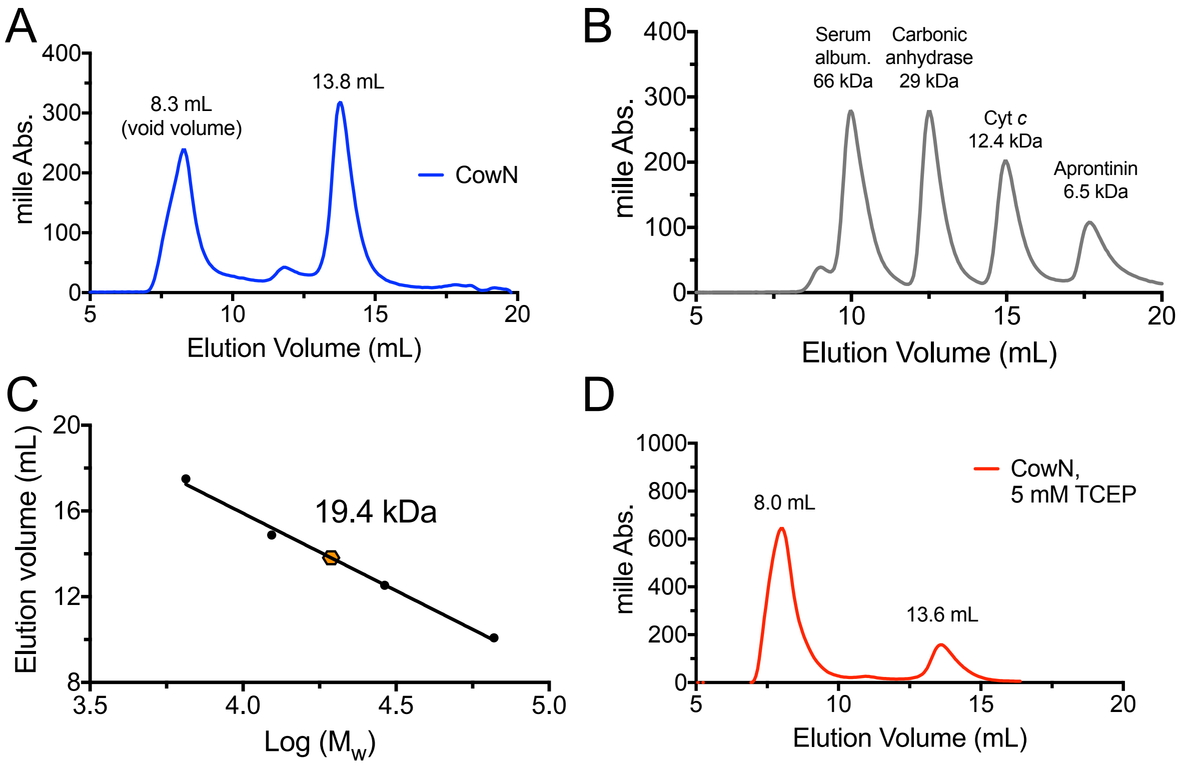

Figure S4. (A) Typical S75 gel filtration chromatogram of CowN in 25 mM HEPES, pH 8, 25 mM NaCl. **(B)** Calibration standards used to calibrate the S75 column. **(C)** Molecular weight determination of CowN. Gel filtration results yield a size of 19.4 kDa, which lies between that of a monomer (13 kDa) and dimer (26 kDa). As discussed in the main text, CowN is most likely a monomer. **(D)** CowN in 25 mM HEPES, pH 8, 25 mM NaCl, 5 mM TCEP (a reducing agent). The relative peak height between the ~ 8 mL and ~13.8 mL peak varies between purifications.

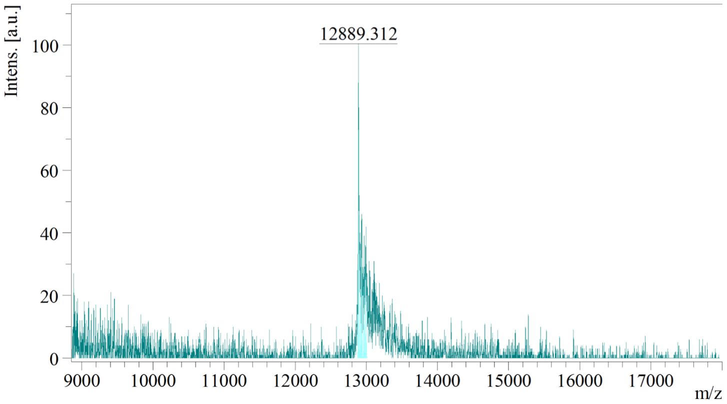

Figure S5. MALDI-TOF of CowN. CowN's experimental mass was determined to be 12,889 g/mol, differing from the expected molecular weight of 13,017 g/mol by a Met residue (within the ± 5 g/mol instrumental error), which suggests the N-terminal Met is cleaved.

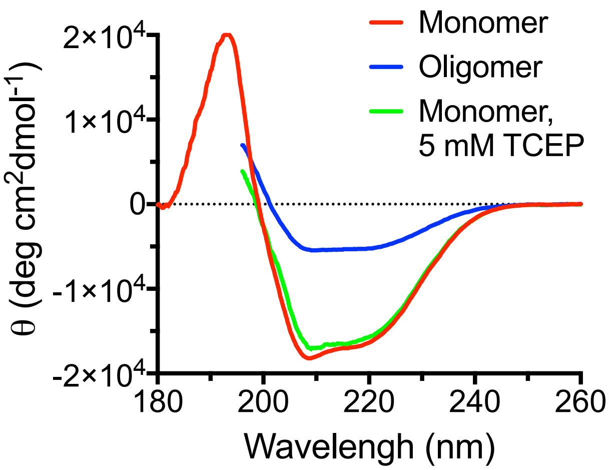

Figure S6. Circular dichroism spectra of CowN monomer, oligomer, and CowN monomer in the presence of 5 mM TCEP, a reducing agent. The spectrum is cut off at 196 nm for CowN oligomer and CowN monomer with TCEP since the signal quality started to deteriorate at that wavelength. Spectra represent the averages of three independent runs.

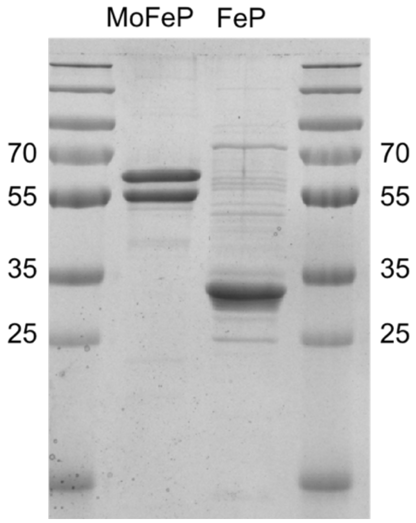

Figure S7. SDS-PAGE of purified nitrogenase component proteins MoFeP and FeP. The two bands in the MoFeP lane represent the α and β subunits of the MoFeP heterodimer. Purity of FeP is similar as in previous reports (2).

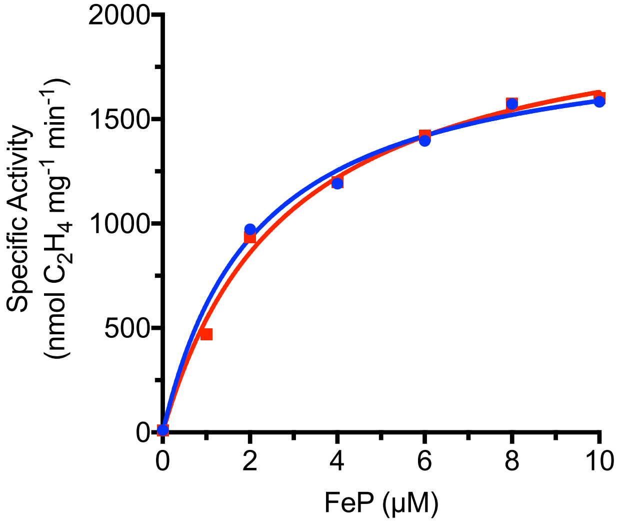

Figure S8. Typical MoFeP activity assays with C_2H_2 as substrate. Maximum specific activity at large excess FeP is approximately 1500 nmol/(mg min)$^{-1}$.

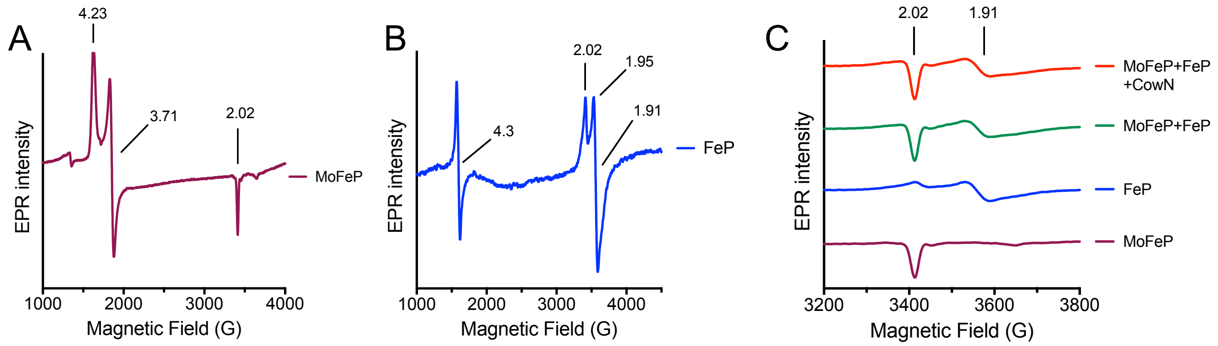

Figure S9. EPR spectra of **(A)** MoFeP and **(B)** FeP. Both MoFeP and FeP are at a concentration of 25 µM. In the FeP sample, the ATP concentration is 10 mM. **(C)** Comparison of the high-field regions of MoFeP alone, FeP alone, FeP and MoFeP, and FeP, MoFeP and CowN under N_2. Values indicated above the spectra correspond to *g*-factors of the features.

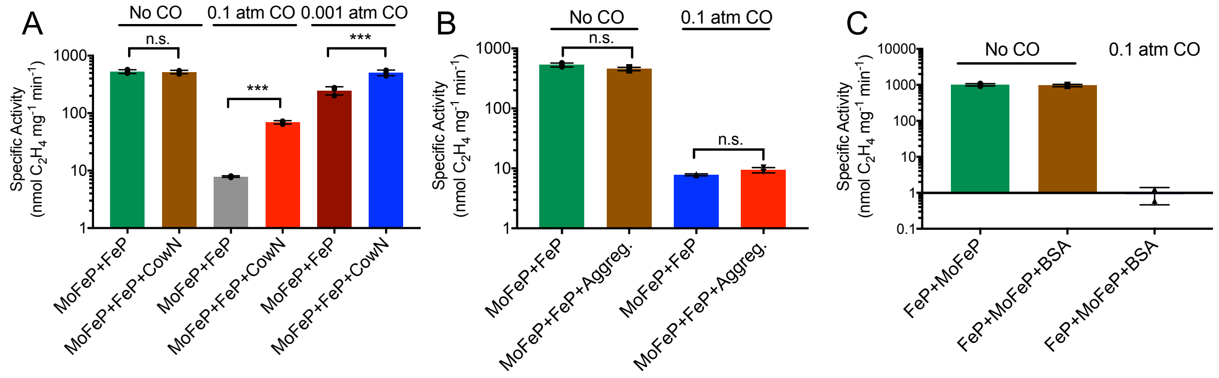

Figure S10. **(A)** and **(B)** display the specific activity on a log scale of main text figures 4A and 4B, respectively. **(C)** Turnover experiments demonstrating that BSA (2 µM) does not protect nitrogenase from CO. Activity differences that are significant to p = 0.001 are denoted by ***. n.s. means no statistical difference.

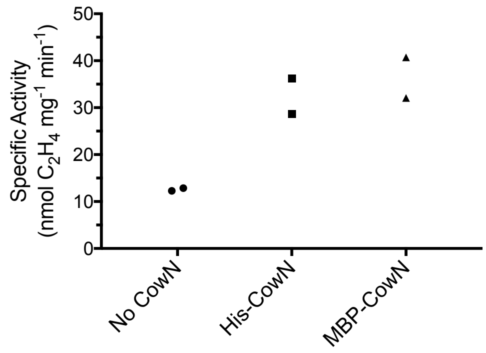

Figure S11. Comparison of nitrogenase protection under 0.1 atm CO by His-CowN and MBP-CowN. Data from duplicate measurements are shown. The experimental conditions are the same is in Figure 4A in the main text except that the FeP:MoFeP ratio is 4:1 instead of 10:1.

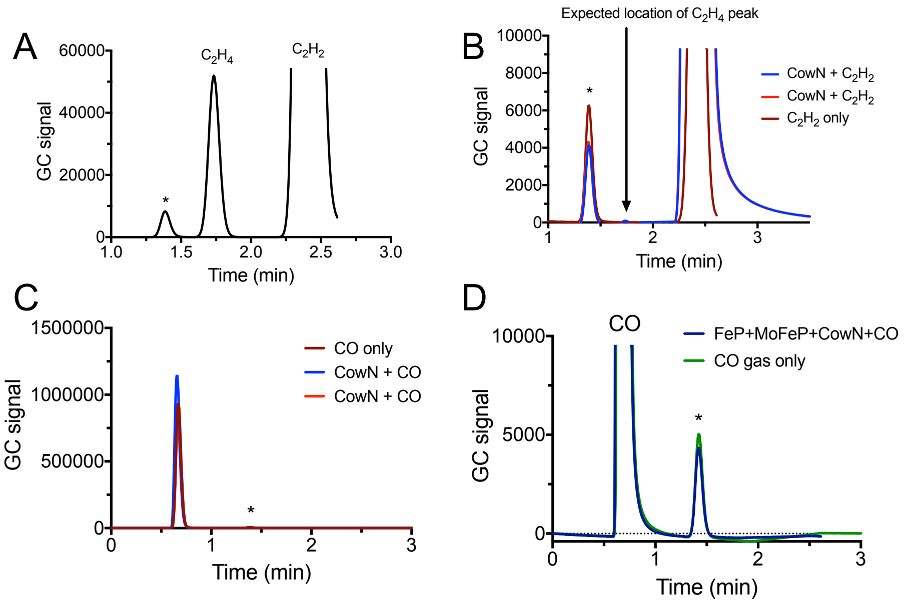

Figure S12. Gas chromatograms demonstrating CowN has no activity towards CO or C_2H_2 on its own and that no CO reduction products are formed during nitrogenase turnover with CowN. **(A)** Typical gas chromatogram for C_2H_2 reduction by nitrogenase without CO, for reference. **(B)** CowN, without nitrogenase, incubated with C_2H_2 compared to a chromatogram of C_2H_2 gas, indicating no new peaks emerge in CowN-only samples, suggesting CowN does not reduce C_2H_2 on its own. **(C)** CowN, without nitrogenase, incubated with CO compared to CO gas only. The chromatograms are similar and no CO is removed/converted by CowN. **(D)** CO reduction experiment with nitrogenase and CowN showing that hydrocarbons, if formed, are below the detection limit. The y-axis is truncated at 10,000 so that the absence of small peaks is clear. CO is detected using a methanizer. The asterisk at 1.4 min denotes a CO_2 impurity present in C_2H_2 and CO gases. The reaction conditions in A-D are the same as in Figure 4A, with the exception that no nitrogenase is present in B and C.

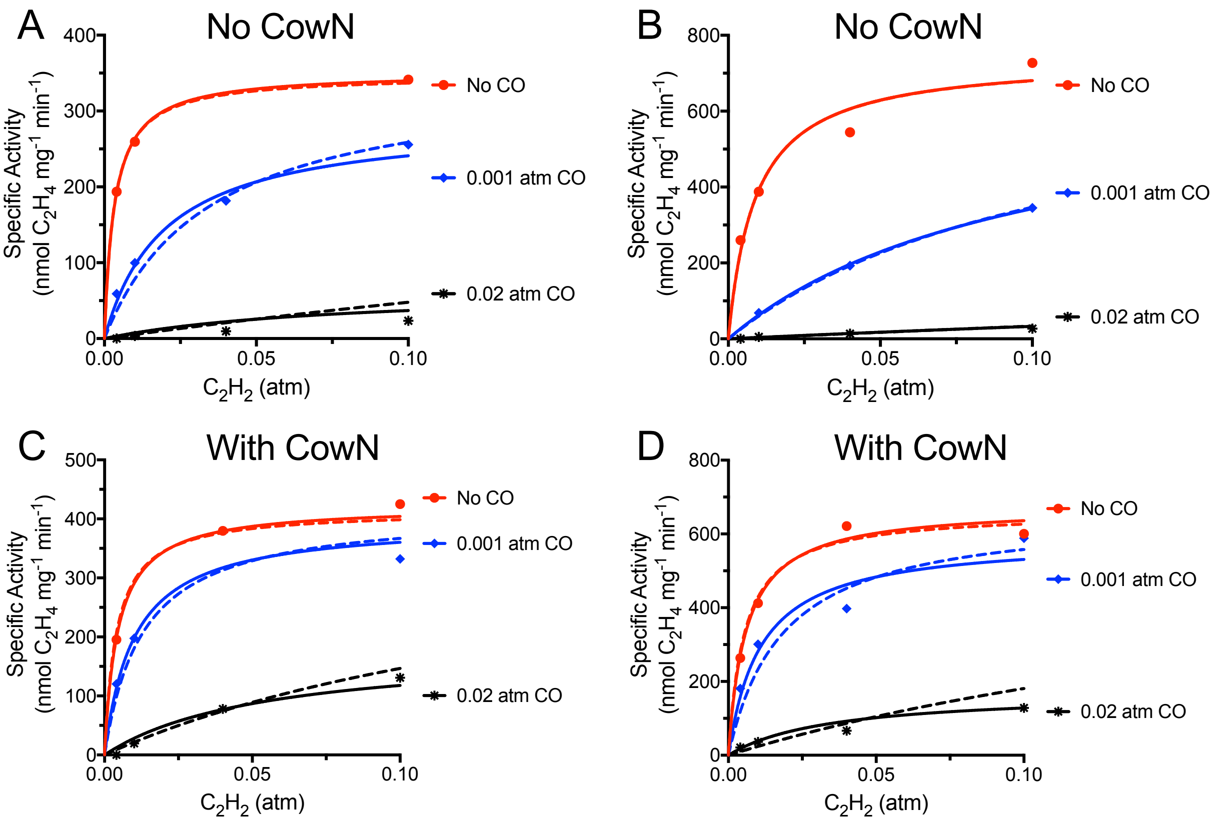

Figure S13. Independent replicates of the CO inhibition experiments shown in Figure 5 of the main text. **(A)** and **(B)** depict CO binding in absence of CowN and **(C)** and **(D)** are conducted in presence of CowN. MoFeP, FeP and CowN concentrations are 0.2 μM, 2 μM and 2 μM, respectively. The data were fit to a mixed inhibitor model (solid lines) and a competitive model (dashed lines). Both models fit equally well to the data in (A) and (B), but the mixed inhibitor model produced the better fit in (C) and (D).

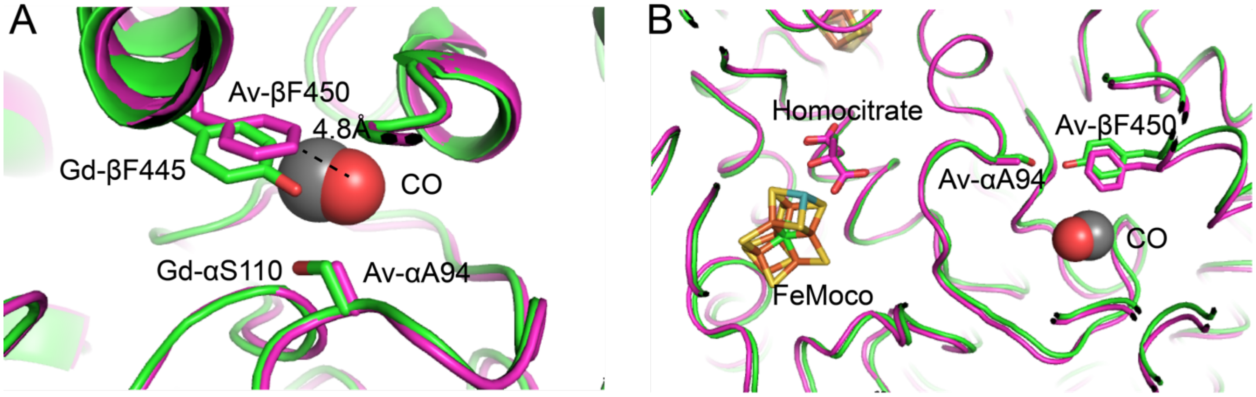

Figure S14. Structural comparison of *Gd*-MoFeP (PDB ID = 4KOH) with a structure of *Av*-MoFeP (PDB ID = 4TKV) in which CO is bound to a putative access channel. *Gd*-MoFeP is colored green and *Av*-MoFeP is magenta. CO is depicted as spheres, where O is red and C is grey. **(A)** Close-up of the CO binding site highlighting the more polar environment in *Gd*-MoFeP. The distance between the edge of *Av*-βF450 and CO is 4.8 Å. **(B)** Cutout through MoFeP to show where CO is located relative to FeMoco.

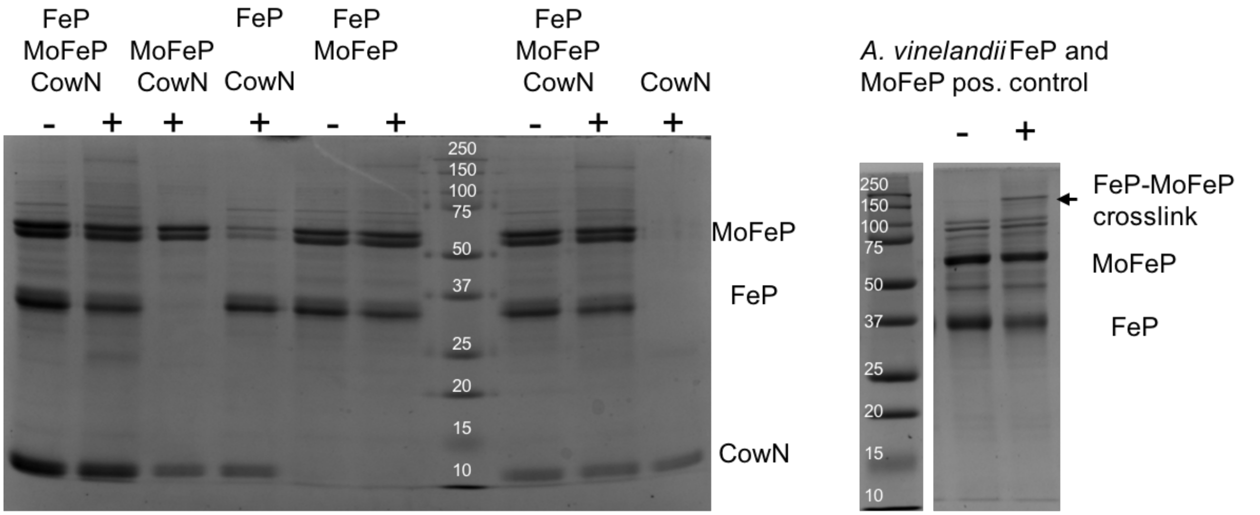

Figure S15. EDC cross-linking experiments between CowN and nitrogenase under nonturnover conditions. The proteins used in each experiment are indicated above each lane. Addition of EDC is indicated by a plus sign. A negative sign means no EDC was added. No new bands appear when comparing EDC cross-linking between FeP and MoFeP with cross-linking between FeP, MoFeP and CowN. Identical results were obtained when the experiment was conducted under turnover conditions and under turnover conditions in the presence of CO. Each experiment was repeated independently, twice. A positive control demonstrating known cross-linking between *Azotobacter vinelandii* FeP and MoFeP is shown on the right. The positive control was conducted under the same experimental conditions as the experiments with *G. diazotrophicus* nitrogenase.

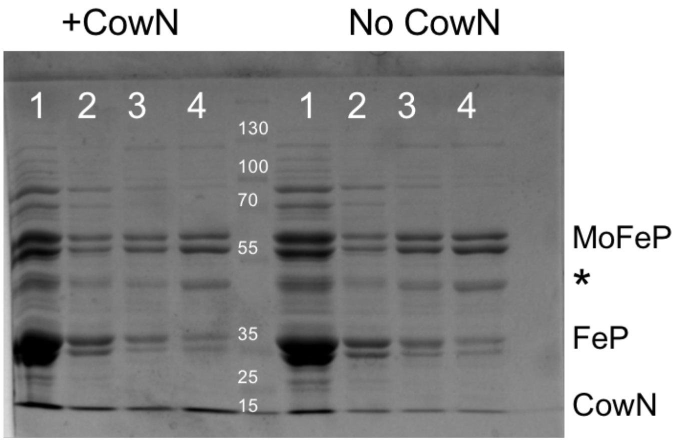

Figure S16. Representative pulldown experiment between CowN and nitrogenase in the presence of CO and under turnover conditions. His-CowN is the bait and nitrogenase the prey. Proteins were pulled down using Ni-NTA beads and the pulled down proteins subjected to four washes with increasing amounts imidazole. Lanes 1-4 contain protein that is present in the first two washes (no imidazole), the third wash (10 mM imidazole) and the final wash (500 mM imidazole), respectively. There is no difference in pulled down protein in the presence and absence of CowN. MoFeP and FeP appear to have nonspecific affinity towards Ni-NTA since some MoFeP and FeP are present in the final wash fraction. Identical results were obtained when the experiment was conducted under nonturnover conditions. The asterisk marks an impurity that was present in the MoFeP sample.

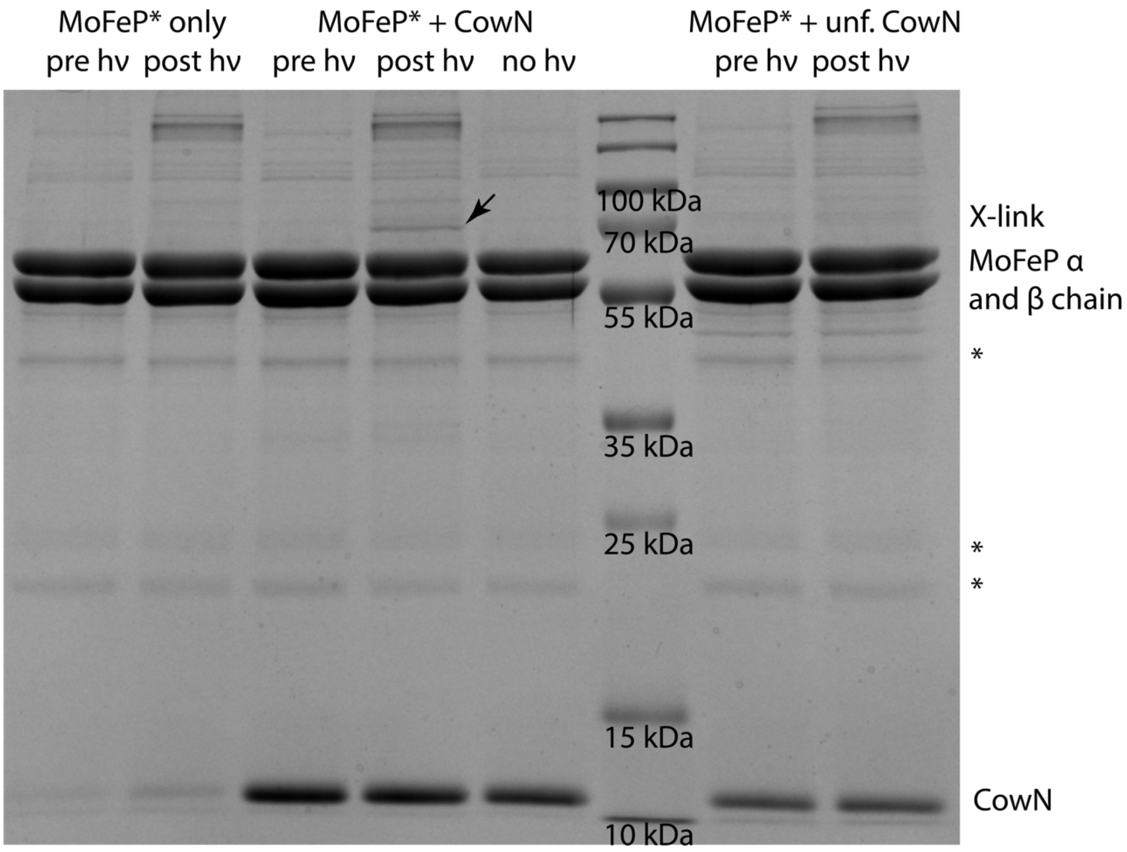

Figure S17. Full gel from main text figure 7. This image includes the MoFeP*-only control that demonstrates that the 70kDa band does not appear without CowN. The cross-link band in the MoFeP*-CowN experiment is marked by an arrow. Asterisks mark impurities.

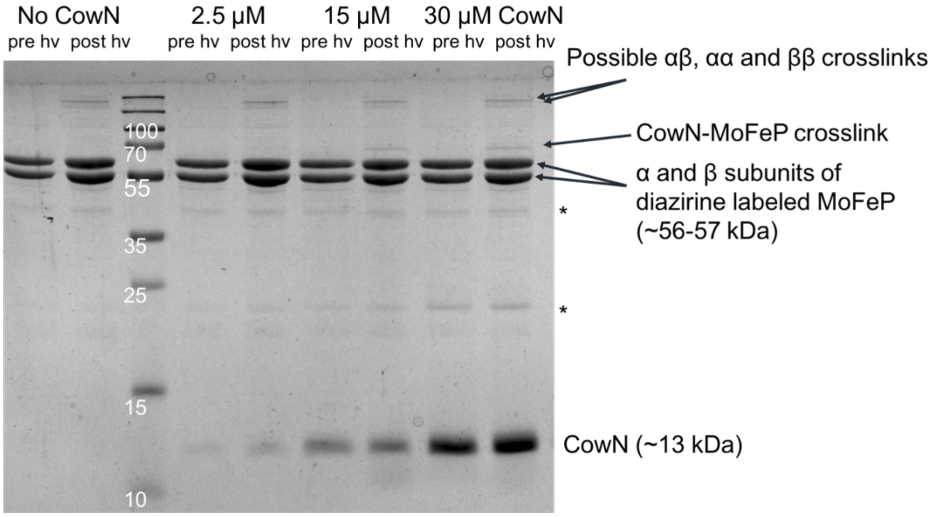

Figure S18. Dose dependence of the 70 kDa cross-link band demonstrating its intensity increases with increasing CowN concentration. Asterisks mark impurities.

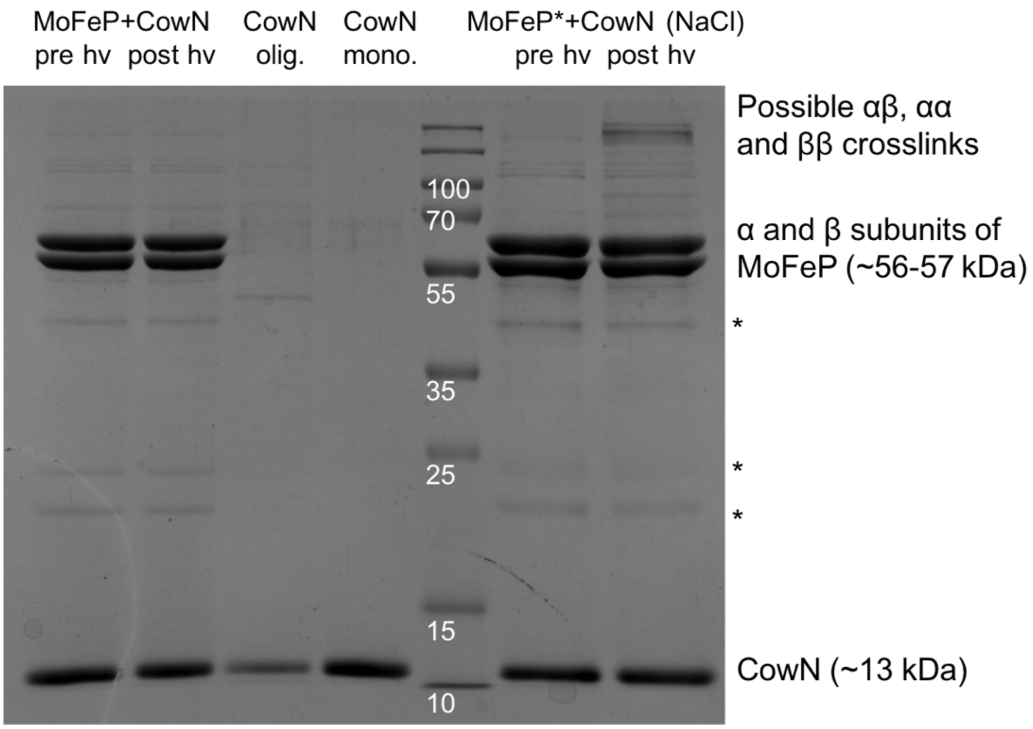

Figure S19. Negative controls demonstrating that unlabeled samples do not form a 70 kDa band after light irradiation and that running the experiment in presence of 500 mM NaCl (instead of the normal 60 mM) disrupts cross-linking. Furthermore, CowN-only samples do not form a 70 kDa band after illumination. Asterisks mark impurities.

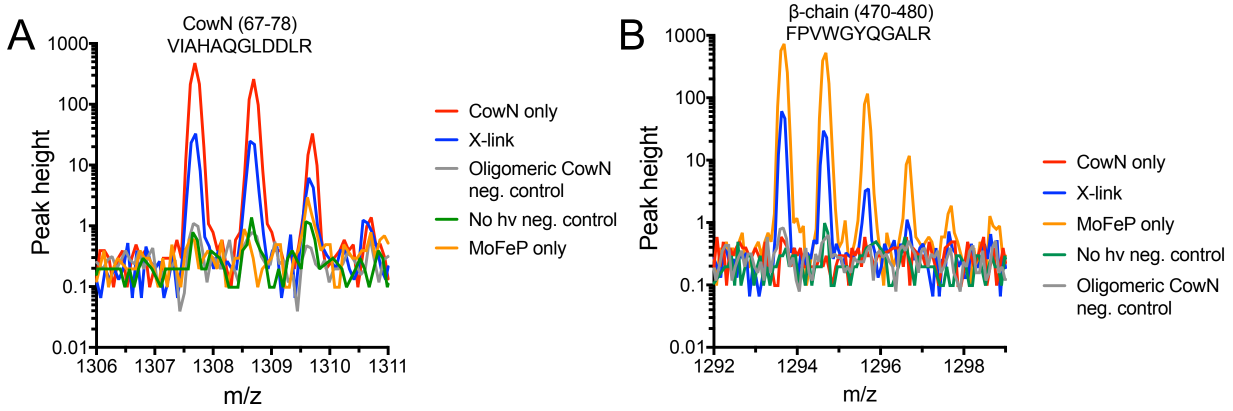

Figure S20. Characteristic MALDI-TOF MS peaks for **(A)** CowN and **(B)** MoFeP demonstrating that CowN and MoFeP are present in the tryptic digest of the 70 kDa band only when the reaction is carried out with MoFeP* and CowN, but not in any of the negative control conditions. CowN-only and MoFeP-only samples represent positive controls obtained from more concentrated CowN-only and MoFeP-only gel slices.

Supporting information references

1. Walsh, I., Minervini, G., Corazza, A., Esposito, G., Tosatto, S. C., and Fogolari, F. (2012) Bluues server: electrostatic properties of wild-type and mutated protein structures. *Bioinformatics* **28**, 2189-2190
2. Owens, C. P., and Tezcan, F. A. (2018) Conformationally gated electron transfer in nitrogenase. isolation, purification, and characterization of nitrogenase from Gluconacetobacter diazotrophicus. *Methods Enzymol.* **599**, 355-386
